# Understanding photosynthetic biofilm productivity and structure through 2D simulation

**DOI:** 10.1371/journal.pcbi.1009904

**Published:** 2022-04-04

**Authors:** Bastien Polizzi, Andrea Fanesi, Filipa Lopes, Magali Ribot, Olivier Bernard

**Affiliations:** 1 Laboratoire de Mathématiques de Besançon, Université Bourgogne Franche-Comté, CNRS UMR-6623, Besançon, France; 2 LGPM, CentraleSupélec, Université Paris Saclay, Gif-sur-Yvette, France; 3 IDP, Université d’Orléans, CNRS, UMR CNRS 7013, Orléans, France; 4 Université Côte d’Azur, INRIA, INRAE, CNRS, Sorbonne Université, BIOCORE, France; 5 LOV-UPMC-CNRS, UMR 7093, Station Zoologique, Villefranche-sur-mer, France; Indian Institute of Technology Bombay, INDIA

## Abstract

We present a spatial model describing the growth of a photosynthetic microalgae biofilm. In this 2D-model we consider photosynthesis, cell carbon accumulation, extracellular matrix excretion, and mortality. The rate of each of these mechanisms is given by kinetic laws regulated by light, nitrate, oxygen and inorganic carbon. The model is based on mixture theory and the behaviour of each component is defined on one hand by mass conservation, which takes into account biological features of the system, and on the other hand by conservation of momentum, which expresses the physical properties of the components. The model simulates the biofilm structural dynamics following an initial colonization phase. It shows that a 75 *μ*m thick active region drives the biofilm development. We then determine the optimal harvesting period and biofilm height which maximize productivity. Finally, different harvesting patterns are tested and their effect on biofilm structure are discussed. The optimal strategy differs whether the objective is to recover the total biofilm or just the algal biomass.

## Author summary

Microalgae have many industrial applications, ranging from aquaculture, pharmaceutics, food industry to green energy. Planktonic cultivation of microalgae is energy-consuming. Growing them under a biofilm form is a new trend with attracting promises. Biofilms are complex heterogeneous ecosystems composed of microorganisms embedded within a self-produced extracellular matrix and stuck to a surface. Most of the studies have focused on bacterial biofilms and knowledge about microalgae biofilms is still very limited. In this paper, we propose a mathematical model describing microalgae biofilm development. We simulate in 1D and 2D the impact of harvesting conditions on biofilm productivity. In agreement with available experimental observations, we find that there exist optimal frequencies and patterns that optimize the productivity. We also show that the optimal conditions differ whether for maximizing the productivity of microalgae or of the whole biofilm.

## 1 Introduction

Microalgae have gained a new biotechnological role in the last decade with applications for food, feed, green chemistry and wastewater treatment [1]. These photosynthetic microorganisms will play a role in the future providing sustainable sources for proteins, pigments, anti-oxidants and many highly valuable products [2]. Some species can store large fractions of triacylglycerol or carbohydrates (after N or P deprivation), which can be turned into biodiesel or bioethanol, respectively. A whelm of research was launched to better understand and exploit this potential renewable source for bioenergy production [3].

These aquatic organisms are mainly cultivated outdoor, in closed photobioreactors or open-ponds where they grow in suspension. The challenge is to convert the flux of solar energy into intracellular organic carbon with maximum efficiency. Microalgae have several advantages compared to terrestrial plants, but the most important feature is that they can grow at a substantially high rate using industrial carbon dioxide and wastewater, with potential productivities one order of magnitude larger than terrestrial plants and without using agricultural land. However, sustainable production with planktonic technologies is very challenging [4]. When growing in suspension, microalgae need continuous agitation to avoid sedimentation and to ensure regular access to light. This requires displacing huge amounts of water, while less than 1% of the liquid mass contains microalgae. Moreover, harvesting algae requires separating them from the medium, and thus energy-demanding operations such as centrifugation or membrane filtration. The price to pay for high productivity is then a high energetic cost for mixing and harvesting which jeopardizes the benefits of microalgae, especially when targeting biofuel production.

More recently, an alternative to suspension growth has been proposed to reduce the need for energy demand. Instead of moving the liquid medium, only the microalgae cells together with their support are set in motion. Several biofilm-based microalgae cultivation systems have been reported, mainly based on lab-scale and indoor conditions for environmental remediation and biofuel production purposes [5, 6]. Various designs have been proposed: the rocker system with polystyrene foam as attaching material [7], the rotating algal disk [8], the rotating rope reactor with spool harvester partially submerged in wastewater [9], the attached cultivation reactor using glass plate and filter paper as a substratum [10] and the conveyer belt [7].

In this new paradigm, microalgae grow within a biofilm, which is an assemblage of microbial cells irreversibly associated with a surface and enclosed in a matrix of extracellular polymeric substances, composed of polysaccharides, proteins, nucleic acids and lipids [11]. Its three-dimensional architecture (spatial arrangement of microorganisms, cells clusters, extracellular polymers and particulates) is inherently heterogeneous, both in space and time, constantly changing due to internal and external processes [12]. Many factors affect biofilm structure, growth and activity, such as nutrient availability, hydrodynamics, cell-cell signalling [13] or even cross-feeding with spacial differentiation [14].

The objective of this work is to represent the development of a microalgal biofilm in a 2D model, to better understand how environmental conditions can affect biofilm structure. Many models have been developed to simulate the structure of bacterial biofilms, but, to our knowledge, only a few studies are available for microalgal biofilms [15]. Taking into account light distribution and inorganic carbon availability in the biofilm is an additional challenge to accurately represent biofilm with photosynthetic microorganisms. Several mathematical models have already been proposed to represent spatial arrangement, growth and metabolic interaction in bacterial biofilms. Multidimensional and multi-species cell-centred models were used in [16, 17] or [18]. However, fluid mechanics effects are difficult to take into account in cellular automata. Hybrid models, coupling some cell-centred approaches with partial differential equations (PDE), have been proposed to improve this point [19].

More physical approaches use continuous models where the biofilm is described as a viscoelastic material that expands in response to the pressure induced by mass exchanges between the biofilm and the surrounding liquid. In some models, biofilm and liquid are separated by a physical interface, which evolution is computed by moving front techniques [20]. Here we use an approach based on mixture theory [21], providing continuous models based on PDEs for multi-component fluids. Mixture models have been successfully used later on for the description of several biological systems and, in particular, for modelling biofilms [20, 22–24].

This study extends the 1D physiological biofilm model [25]. In this approach, we consider several physiological processes which affect intracellular dynamics, such as photosynthesis, organic carbon intracellular storage, extracellular matrix excretion, and mortality. The associated kinetic rates are functions of intracellular variables and dissolved compounds. In particular, we propose a structured description of the microalgae growth, based on the consideration of intracellular reserves triggering the processes of growth, respiration and polymer excretion. We consider separately the intracellular storage of carbon (lipids and carbohydrates) and the functional part of the cells (proteins, membranes, DNA, etc …). Here we extend this model to 2D, to assess the impact of environmental conditions, especially harvesting, on biofilm structure and growth and to better describe the structures appearing along the biofilm development.

## 2 Model

### 2.1 Physiological model principles

This section recalls the model principles as they were presented in details in [25] for the 1D version. The biological model is a trade-off between macroscopic models which do not account for intracellular physiology and metabolic models of high complexity, which would be difficult to embed in a physical structure. The model was designed to accurately describe the accumulation of carbon reserve (lipid, carbohydrate, EPS) in the cell and its production within the Extra-Cellular Matrix (ECM). To our knowledge, it is the first model for algal biofilm combining a physiological description with the spatial dynamics and structuration of the biofilm architecture.

Microalgae are embedded in the biofilm within a self produced extra-cellular matrix. Photosynthesis takes place in the chloroplasts, using inorganic carbon (mainly CO_2_) as substrate. Extra-cellular matrix is made of dead cells and excreted extra-polymeric substances and proteins [26].

In line with other models [27, 28], the microalgal biomass is split into functional biomass and a pool of carbon storage. The functional biomass gathers biosynthetic molecules such as proteins, nucleic acids and structural material. The pool of carbon storage is made of lipids and carbohydrates. The mathematical model therefore contains four constituents: pool of carbon storage (**A**), functional biomass (**N**), extra-cellular matrix (**E**) and liquid phase (**L**). Note that the sum **M** = **A** + **N** represents the microalgal biomass. We also consider three dissolved components in the liquid phase: inorganic carbon (**C**) (mainly CO_2_ and bicarbonate), oxygen (**O**) and nitrate (**S**, substrate).

It is important to understand that the liquid phase **L** represents the free water trapped into the biofilm, but that all the components are assumed to be linked to a liquid phase. We consider that the components **A**, **N** and **E** include 90% of the associated liquid inside the cellular membrane.

Photosynthesis consumes carbon dioxide and water, releases oxygen and produces carbohydrate. The photosynthesis rate increases and saturates with carbon dioxide and free water. Photoinhibition is also included using Peeters-Eilers model [29] and oxygen [30] can inhibit photosynthesis at high concentration.

In the opposite, cells use dissolved oxygen to oxidise the carbohydrate in the respiration process, which releases carbon dioxide and water.

Finally, a fraction of the extra-cellular matrix is composed of EPS excreted by the microalgae. If nitrogen is available, cells synthesise the components of functional biomass and grow. In case nitrogen limitation induces an imbalance between nitrogen and carbon fluxes, microalgae start to release exopolysaccharides [31], but also a fraction of the functional biomass, composed of proteins. Dead microalgae also contribute to the extra-cellular matrix. Mortality rate is supposed to depend on dissolved oxygen concentration in the following way: when oxygen concentration becomes smaller than a subsistence level, mortality is enhanced. Moreover, at high oxygen concentration, oxygen free radicals are generated, inducing cell mortality [30, 32].

**Fig 1:**
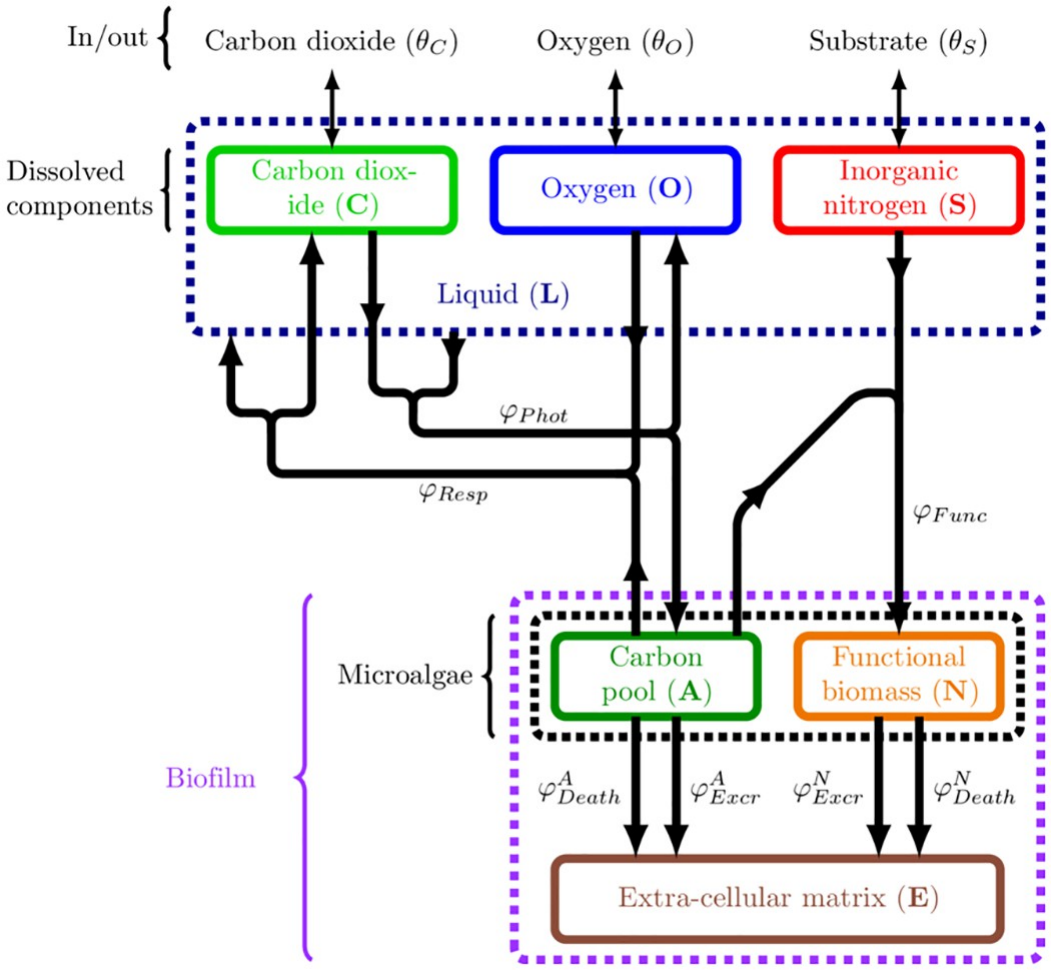
Schematic representation of the biological model scheme, including the external supplies and the metabolic pathways. The arrows represent the mass exchanges induced by biochemical reactions between the components which are represented by the rectangles.

Fig 1 presents an overview of the forementioned mass fluxes. The pseudo stoichiometric coefficient associated to photosynthesis (resp. respiration, functional biomass synthesis, EPS excretion and microalgae death) for component *ϕ* is denoted by $\eta_{Phot}^{\phi }$ (resp. $\eta_{Resp}^{\phi }$, $\eta_{Func}^{\phi }$, $\eta_{Excr}^{\phi }$ and $\eta_{Death}^{\phi }$); here, *ϕ* stands for one of the components *ϕ* ∈ {*A*, *N*, *E*, *L*, *S*, *C*, *O*}. The pseudo stoichiometric coefficient values are displayed in Table B in S3 Text. We also call *φ*_*Phot*_ (resp. *φ*_*Resp*_, *φ*_*Func*_, *φ*_*Excr*_, *φ*_*Death*_) the photosynthesis (resp. respiration, functional biomass synthesis, EPS excretion and microalgae death) rate. We present in S1 Text the complete expression of all these functions, see [25]. Parameter values are also the same as in [25] and are given in Tables A-F in S3 Text.

### 2.2 Physical model

Biofilms are multi-phasic heterogeneous ecosystems and their physical properties, especially for micro-algae biofilms are not well understood. Modelling such complex systems and taking into account this heterogeneity is therefore challenging. For example, the biofilms its self is a visco-elastic material [33, 34] whereas the surrounding bulk liquid is not. Nevertheless, although micro-algae biofilms differ from bacterial biofilms on the metabolism point of view, they share similarities in their mechanical properties. Indeed, the mechanical properties of biofilms mainly come from their structure: both micro-algae and bacterial biofilms are made of close-packed micro-organisms within a self produced extra-polymeric matrix. Thus, the formalism developed for bacterial biofilms can be adapted to micro-algae biofilms.

As mentioned in the introduction, different approaches have already been proposed to represent spatial arrangement, growth and metabolic interactions in biofilms. Among these approaches, PDE based models are well suited to account for physical properties such as fluid mechanics effects. The mixture theory framework [35], which generalises Euler equations, can combine continuum theory for the motion and deformation of solids and fluids with general principles. Therefore, it is well adapted to account for heterogeneity within biofilms.

More precisely, we consider two kinds of components, namely biofilm constituents (ie. carbon pool, functional biomass and extra-cellular matrix) and liquid on the one hand and dissolved components (eg. oxygen) on the other hand, see previous subsection. All the model variables depend on time and space.

We describe first the equations for the three biofilm constituents and the liquid. Following [22], those components are described through their mass balance equation and their force balance equation, which are all described in details in S1 Text.

The mass balance equations take the form of advection-reaction equations. They gather all the biological features of the model through the reactions terms that describe the mass fluxes between components. The local composition affects the bioreaction rates, thus the reaction terms are nonlinear functions that account for the different limitation and inhibition mechanisms. The force balance equations gather the physical properties of the system, which are hardly known. Therefore, following [22], we only consider the hydrostatic pressure, the elastic tensor, the friction between phases and the momentum supply induced by mass exchanges. Other processes like aggregation or osmotic pressure could also be included; for example in [36] Flory-Huggins forces are considered, leading to mushroom-like structure formation. Since there is little experimental work to support the underlying hypotheses on the involved forces in the case of microalgae biofilms, only the behaviour of the model, like structure formation, can justify the choice of mechanical modelling. Here structure formation matching experimental datas are observed with only the forces mentioned above.

Since the model is based on mixture theory, each of these four components is described by its volume fraction which represents the percentage of the volume occupied locally by the component. This formalism induces a volume constraint, which says that the whole volume is occupied by the four constituents. This constraint can be expressed equivalently as a non-homogeneous incompressibility constraint, in the spirit of Navier-Stokes incompressibility constraint.

Let us now detail the equations for the dissolved components. Inorganic carbon **C**, oxygen **O** and nitrate **S** are dissolved within the liquid phase. Thus these components are advected by the liquid and there is no momentum equation for them. As for the other components, the reaction terms involve non-linear functions to account for the limitations in mass exchange fluxes. In addition, these components can diffuse at a constant rate. Therefore, the mass balance equations for dissolved components take the form of advection-reaction-diffusion equations. Finally, the external supply of inorganic carbon and oxygen is modelled using Henry’s balance law, whereas the external supply of nitrate comes from the boundary condition. Here again, all the details are presented in S1 Text.

To sum up, the general form of this model is the same as the model in [22], but it differs on several points. First, the physiology in the mass balance equations is described with many more details, which complicates the incompressibility constraint. Then, we consider three distinct velocities in the force balance equations: **v**_*M*_ for the microalgae, **v**_*E*_ for the extra-cellular matrix and **v**_*L*_ for the liquid phase. Finally, we couple the mixture model with complementary mass balance equations detailing the evolution of the components dissolved in liquid. The numerical scheme used to discretize the full system of equations is explained in S4 Text.

Here, the model is simulated in two dimensions, but the extension in this paper and the general equations proposed in S1 and S2 Text are also valid in three dimensions.

## 3 Results and discussion

The numerical results presented here after are done with Matlab 2018b. The code is available at: https://plmlab.math.cnrs.fr/polizzi/photosynthetic-biofilm-simulations/.

### 3.1 Structure dynamics: Starting from a spot colony

**Fig 2:**
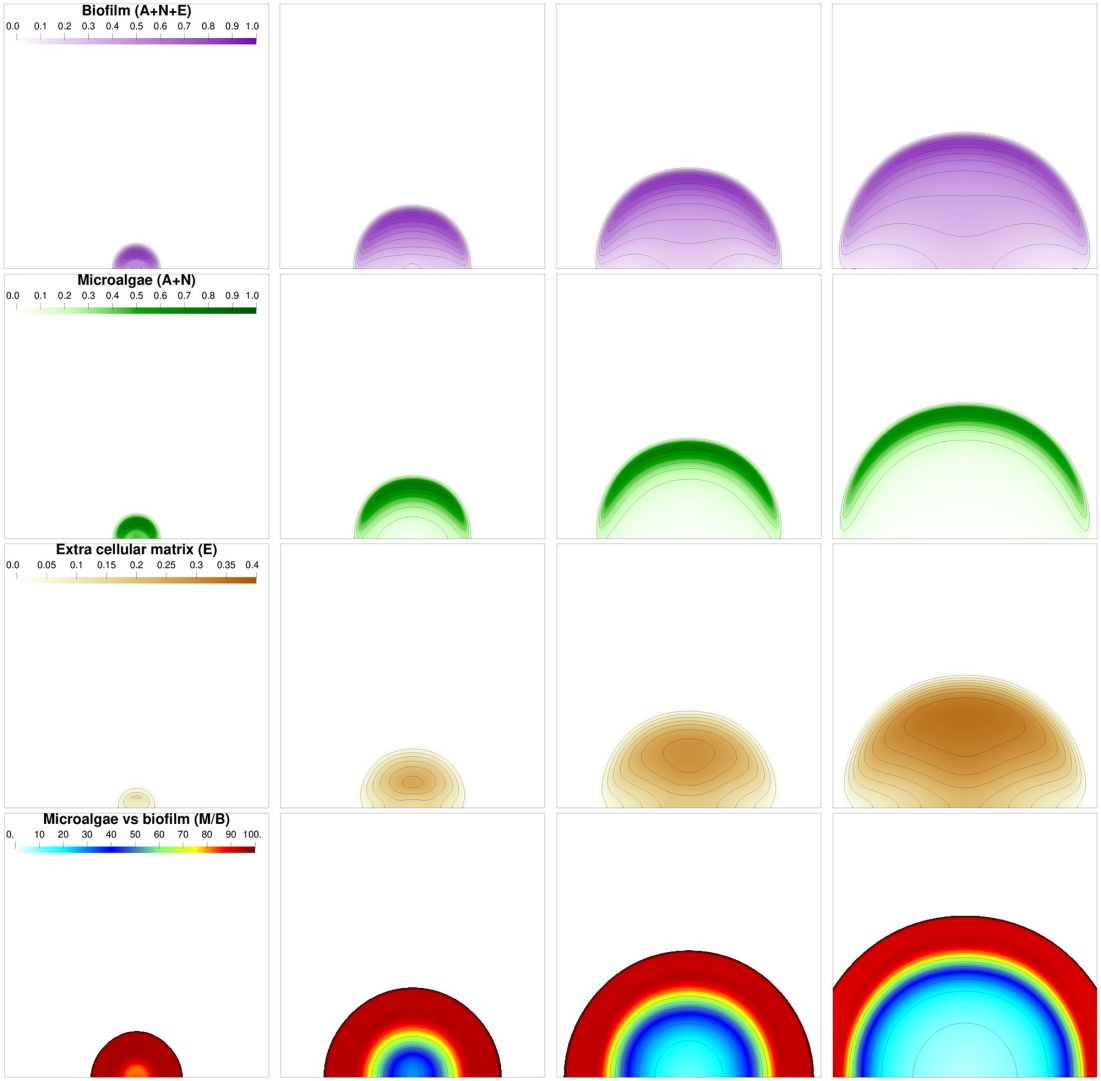
Biofilm volume fractions and composition at different times: *t* = 5, *t* = 15, *t* = 25 and *t* = 35 days from left to right. The first row represents the whole biofilm volume fraction *B* = *A* + *N* + *E*. The second row represents the microalgae volume fraction *M* = *A* + *N*. The third row represents the extra cellular matrix volume fraction *E*. Finally the last row represents the ratio of living biomass over the whole biomass, namely 100 × *M*/*B*.

**Fig 3:**
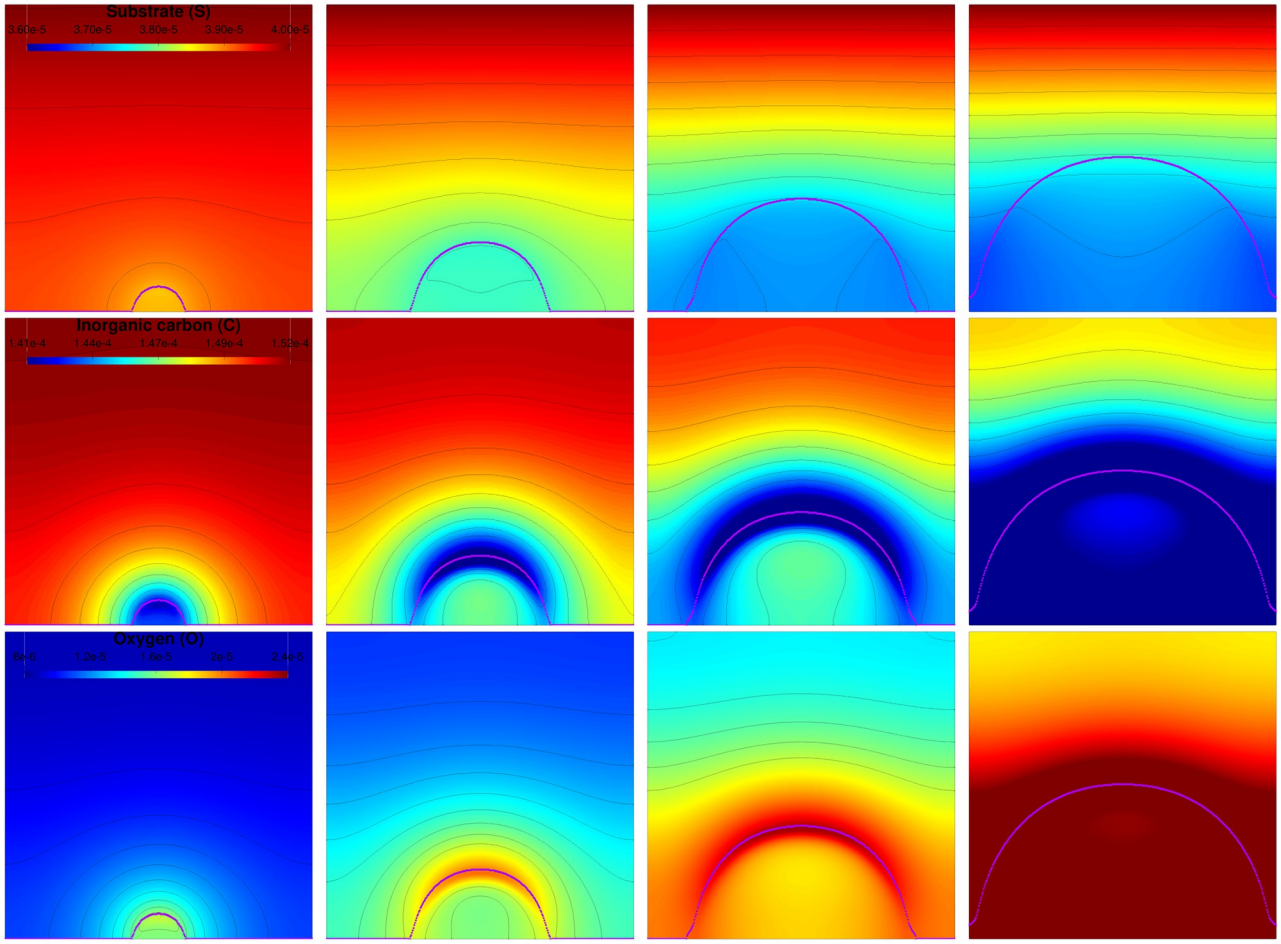
Distribution of the dissolved components for a biofilm starting from a single spot colony. The inorganic nitrogen concentration (first line), inorganic carbon distribution (second line) and oxygen distribution (third line) are represented at times *t* = 5, *t* = 15, *t* = 25 and *t* = 35 days (from left to right). The purple dotted line represents the biofilm front defined as the largest value for the biofilm gradient.

First, we consider the development of a biofilm originating from a single colony. The initial distribution is defined in S3 Text section 1. Fig 2 shows the simulated biofilm growth. The microalgae distribution (for **A** and **N**) takes a moon crescent shape on the front while the extra-cellular matrix E is accumulating behind it. A similar development pattern has been observed in monospecific microalgae biofilms produced by *C. autotrophica, P. purpureum* and *C. closterium* [37–39]. It is interesting to notice that such a spatial separation among biofilm components (i.e. cells on top and matrix at the bottom) could strongly impact the mechanical properties of biofilms. For instance, erosion tests applied on bacterial and microalgal biofilms demonstrated that the bottom layers of the biofilms are often more cohesive than their top layers, this cohesion gradient has been correlated to EPS distribution [37–39]. The growth of the biofilm seemed to be mainly driven by light, CO_2_ and O_2_ gradients, which were more available on the top of the biofilm and decreased quickly within it, as shown on Fig 3. The distribution of the dissolved components, at different times, points out that they develop faster in this front area. Consequently, the volume fraction of microalgae on the front is increasing and the biofilm takes the characteristic form of a mushroom, as already described in the literature [24] and observed experimentally for bacterial biofilms [40, 41]. This structure appears after few days and is completely established at day 15 according to the second row in Fig 2 displaying microalgae volume fraction (*M* = *A* + *N*).

### 3.2 Analysis of the main mass fluxes

**Fig 4:**
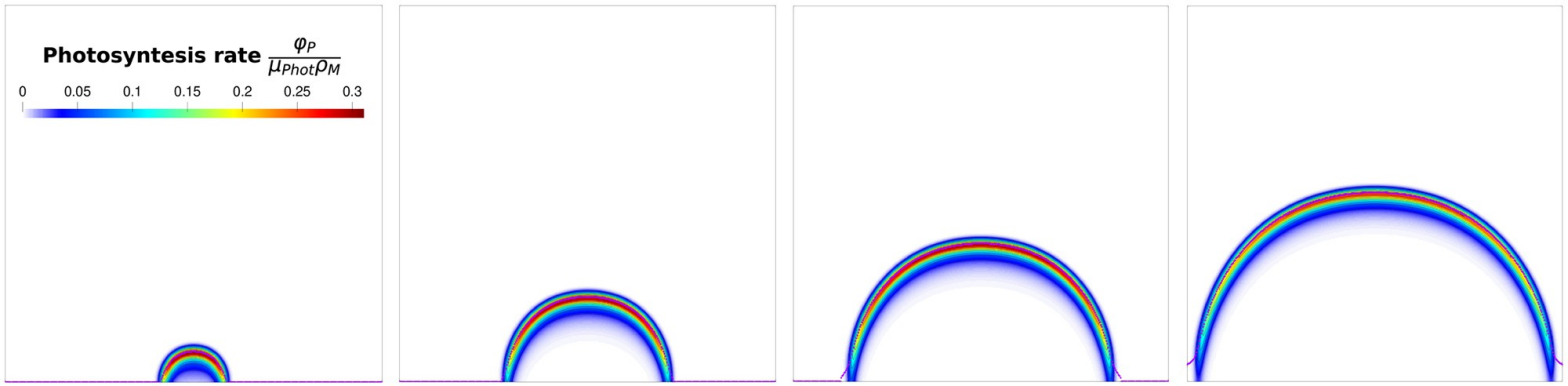
Photosynthesis rate in the biofilm (*φ*_*Phot*_, see S2 Text for the detailed mathematical expression) at times *t* = 5, *t* = 15, *t* = 25 and *t* = 35 days for a biofilm starting from a single spot colony. The purple dotted line represents the biofilm front.

**Fig 5:**
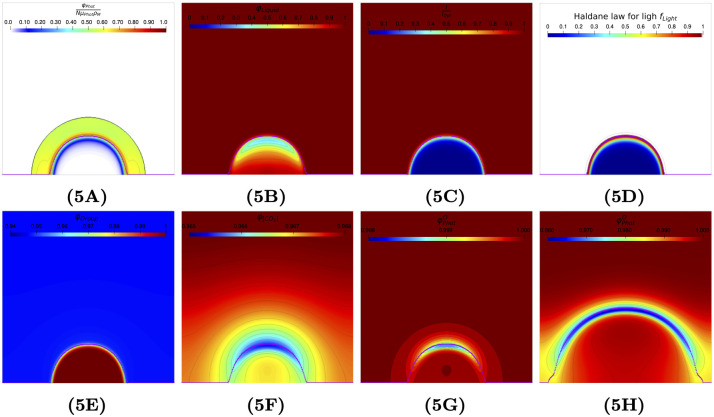
Relative contributions to photosynthesis rate of different terms considered at time *t* = 15 days (except Fig 5H at time *t* = 30 days); see S2 Text for the expression of these terms. Fig 5A: Normalized photosynthesis rate $\frac{\varphi_{Phot}}{N\mu_{Phot}\rho_{M}}$. Fig 5B: Photosynthesis limitation by liquid *f*_*Liquid*_. Fig 5C: Relative light intensity $\hat{I}$. Fig 5D: Photosynthesis limitation by light *f*_*Light*_ using Haldane law. Fig 5E: Photosynthesis limitation by functional biomass quota *f*_*Droop*_. Fig 5F: Photosynthesis limitation by inorganic carbon $f_{\left[CO_{2}\right]}$. Fig 5G: Photosynthesis limitation by oxygen *f*_*Oxy*_ at time *t* = 15 days. Fig 5H: Photosynthesis limitation by oxygen: *f*_*Oxy*_ at time *t* = 30 days.

Biofilms are characterized by steep physical and chemical gradients that define the vertical spatial organization of cell layers in term of activity and physiology [42]. Therefore, it is of paramount importance to relate such gradients to cell activity in order to spatially assess processes such as growth and death within a biofilm. Fig 4 represents the photosynthesis rate in the biofilm (ie. *φ*_*Phot*_, see S2 Text for the detailed mathematical expression) at times *t* = 5, *t* = 15, *t* = 25 and *t* = 35 days for a biofilm starting from a single spot colony (see Sec. 3.1). It is interesting to note that the thickness of the active biofilm (defined as the area where the photosynthesis rate is larger than 1% of its maximal) reaches a constant value of 75 ± 7.5 *μ*m after 5 days. This means that the superficial layers of the biofilm are the most photosynthetically active, as supported by several experimental studies on phototrophic biofilms [43–45]. Typically, the factor driving the vertical distribution of the active layers in photosynthetic biofilms is light, which is imposing the extent of the euphotic zone [43]. Accordingly, Fig 5C shows that the localization of the cells at the top layers drastically attenuate the available light over the depth of the biofilm. Thus, the biofilm growth resulted to be strongly light limited, as shown in Fig 5D representing the light effect on the photosynthesis rate (*ϕ*_*Phot*_). Our simulation also highlighted that low liquid content constrains the photosynthesis rate (Fig 5B). It is worth recalling that variable *L* represents the free water trapped within the biofilm, which can become limiting, while the chemically combined water is not affected. Around and below the biofilm front (ie. purple dotted line), the free liquid volume fraction becomes very low (below 20%), then the term *φ*_*Liquid*_ is around 0.4 meaning that photosynthesis rate is reduced by 60%. [46] found that after a decrease by 15% of relative air humidity (from 100% to 85%), the photosynthetic efficiency of aeroterrestrial microalgae biofilms dropped to zero and the amount of non-growing algae increased, confirming the importance of water availability on photosynthetic biofilms. Fig 5E and 5F show that there is no marked nitrate and CO_2_ limitation in the biofilm for the considered growth conditions. Indeed, those substrates penetrate the biofilm and the saturation level in the medium guarantees that there is no limitation, see Table C in S3 Text. Similarly, [37] found that the diffusion coefficient in *C. vulgaris* biofilms did not correlate with changes in their architecture, indicating that pores and channels might ensure a continuous supply of nutrients within the core of a biofilm. According to Fig 5G, until day 15, no inhibition by oxygen is expected to occur. However, for longer times, the oxygen accumulation around the biofilm front eventually induces photosynthesis inhibition and cell death (see Fig 5H at t = 30 days). Indeed, in photosynthetic biofilms and mats the presence of high oxygen concentrations can inhibit photosynthesis by stimulating the oxygenase activity of RuBisCo (i.e. photorespiration) or by inducing ROS generation [47].

**Fig 6:**
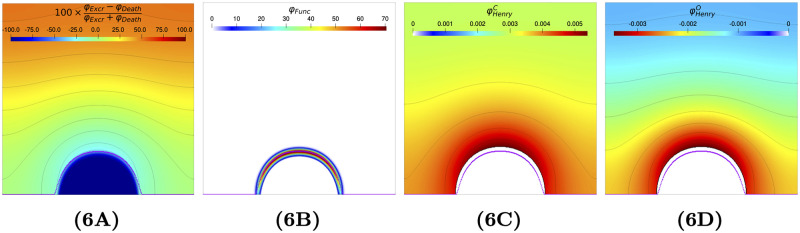
Relative difference between excretion and death rates, functional biomass production, inorganic carbon transfer rate and oxygen ransfer rate at day 15 (see S2 Text for the expression of these terms). Fig 6A: Relative difference (Δ in %) between fluxes for ECM excretion and death. Fig 6B: Functional biomass production rate *φ*_*Func*_. Fig 6C: Inorganic carbon supply $\varphi_{Henry}^{C}$. Fig 6D: Oxygen supply $\varphi_{Henry}^{O}$.

Fig 6A represents the relative difference (Δ in %) between the cell excretion and cell death:

$$\begin{array}{c} \Delta =100\times \frac{\varphi_{Excr}-\varphi_{Death}}{\varphi_{Excr}+\varphi_{Death}} \end{array}$$

with $\varphi_{Excr}=\varphi_{Excr}^{A}+\varphi_{Excr}^{N}$ and $\varphi_{Death}=\varphi_{Death}^{A}+\varphi_{Death}^{N}$.

The biofilm frontier is represented in Fig 6 by a purple curve. It turns out that the ECM production inside the biofilm (below the purple line) mainly comes from the death process as described for bacterial biofilms where volumetric growth resulted from the accumulation of inactive organic matter constituted by dead cells and EPS [48]. In the external layer of the biofilm, death and excretion equally contribute to ECM creation. In these top layers, where oxygen concentration and light are high, excretion could be interpreted as an acclimation mechanism to allow excess absorbed energy to flow avoiding the over-reduction of the photosynthetic machinery [49]. Only microalgae from the outer layer of the biofilm are active and produce functional biomass (Fig 6B).

### 3.3 Structure formation: Starting from multi-colonies

**Fig 7:**
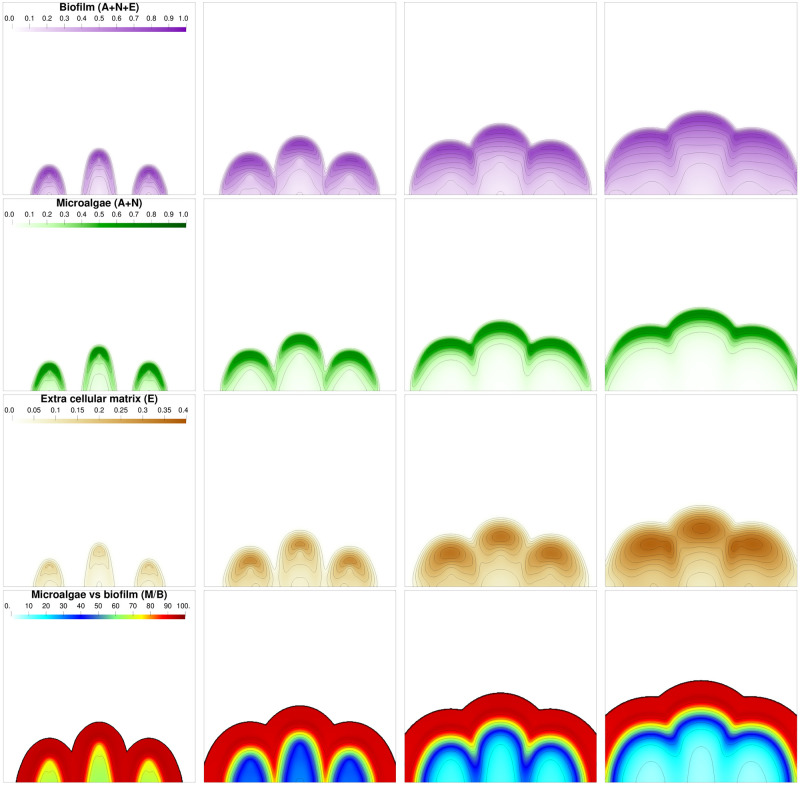
Biofilm volume fractions and composition at different times: *t* = 5, *t* = 10, *t* = 15 and *t* = 20 days from left to right. First row: whole biofilm volume fraction *B* = *A* + *N* + *E*. Second row: microalgae volume fraction *M* = *A* + *N*. Third row: extra cellular matrix volume fraction *E*. Last row: ratio (%) of living biomass over the whole biomass *M*/*B*.

**Fig 8:**
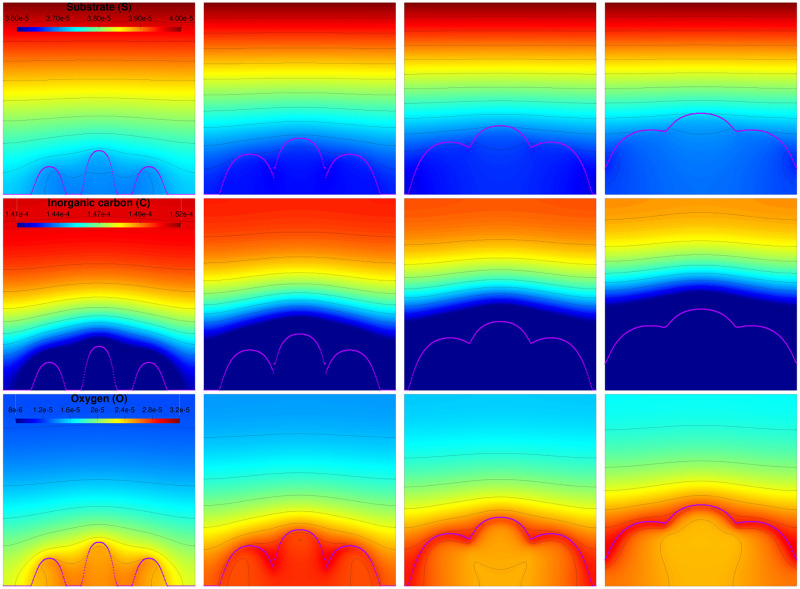
Concentration of the dissolved components at times *t* = 5, *t* = 10, *t* = 15 and *t* = 20 days for a biofilm starting from a three spot colony. First line: substrate distribution (**S**). Second line: inorganic carbon distribution (**C**). Third line: oxygen distribution (**O**). The purple dotted line represents the biofilm outer contour defined as the largest value for the biofilm gradient.

The second simulation starts from an initial distribution made of three algal colonies (see S3 Text). The different biofilm components and the dissolved elements are represented along the biofilm growth in Figs 7 and 8, respectively. Until *t* = 10 days colonies grow separately and are mostly made of microalgae. Microalgae on the front have a better access to nutrients and light so their growth is faster, as previously stated [50]. Consequently the biofilm volume fraction is higher on the front than inside the biofilm which leads to a mushroom shape for each bump at t = 20 days. Then the three bumps merge and a joined front made of microalgae develops. At a macroscopic level, it means that the biofilm surface roughness progressively decreases with time. This result is supported by experimental observations for different species [51]. Finally, once the multiple initial colonies have merged, the growth dynamics is very similar to that of a single colony, with similar determinants driving biofilm development. Although in our simulation the colonies merge to form a sole element as reported for bacterial biofilms [52], in multi-species aggregates, mechanisms such as competition may occur inducing spatial segregation [53]. Future developments introducing in the model chemical signalling or processes such as competition and cooperation could be of interest to understand the microbial spatial organization in complex photosynthetic biofilms.

### 3.4 Impact of harvesting on productivity

#### 3.4.1 Numerical strategy

Two-dimensional numerical simulations are expensive in terms of CPU resources (typically, the simulations presented in the previous section take 2 to 3 weeks on a *Xeon SP Gold 6148 @ 2.40GHz*). Therefore, it is out of reach to assess the impact of harvesting strategies directly on the two-dimensional model. For an initial condition uniform in space, the model can be approximated by a 1D model, which computations are a range of order faster. In a first step we validate this one-dimensional approximation and show that for spatial uniform initial condition the results are in good agreement with the two-dimensional simulations.

Then we use the 1D model to run an extensive numerical analysis to assess the effect of harvesting height and harvesting period.

Once the main parameters affecting productivity have been identified, two-dimensional simulations are run with the third objective to test different scrapping patterns and determine their effect on productivity.

#### 3.4.2 Validation of 1D estimates for uniform initial conditions

To validate the 1D productivity estimates for uniform harvest, we compare a set of 1D and 2D simulations. Since 2D numerical simulations are highly computational demanding, the comparison has been made for the four representative situations listed in Table 1. Moreover, the tolerance used to estimate if the stationary regime is reached is ten times larger for 2D simulations than for 1D simulations. The results are summarized in Table 1. Accordingly, the relative productivity difference between 1D and 2D simulations remains under 1% except for one case. Indeed, for the simulation corresponding to harvest every 4 days at 350 *μm* the relative difference is about 3.26%. In this case, the stationary regime is not perfectly reached in the 2D simulation; indeed, the tolerance value we choose induces 13 harvest periods for the 2D simulation instead of 16 harvest periods for the 1D simulation, which explains this discrepancy.

**Table 1:** Relative productivity difference between 1D and 2D numerical simulations for the whole biofilm (ie. A+N+E) in the case of a uniform harvest.

Harvest period (days)	4	6.5	9	9
Harvest height (*μm*)	350	350	75	325
Relative difference (%)	3.26	0.35	0.01	0.34

### 3.5 Impact of height and harvest period on productivity

**Fig 9:**
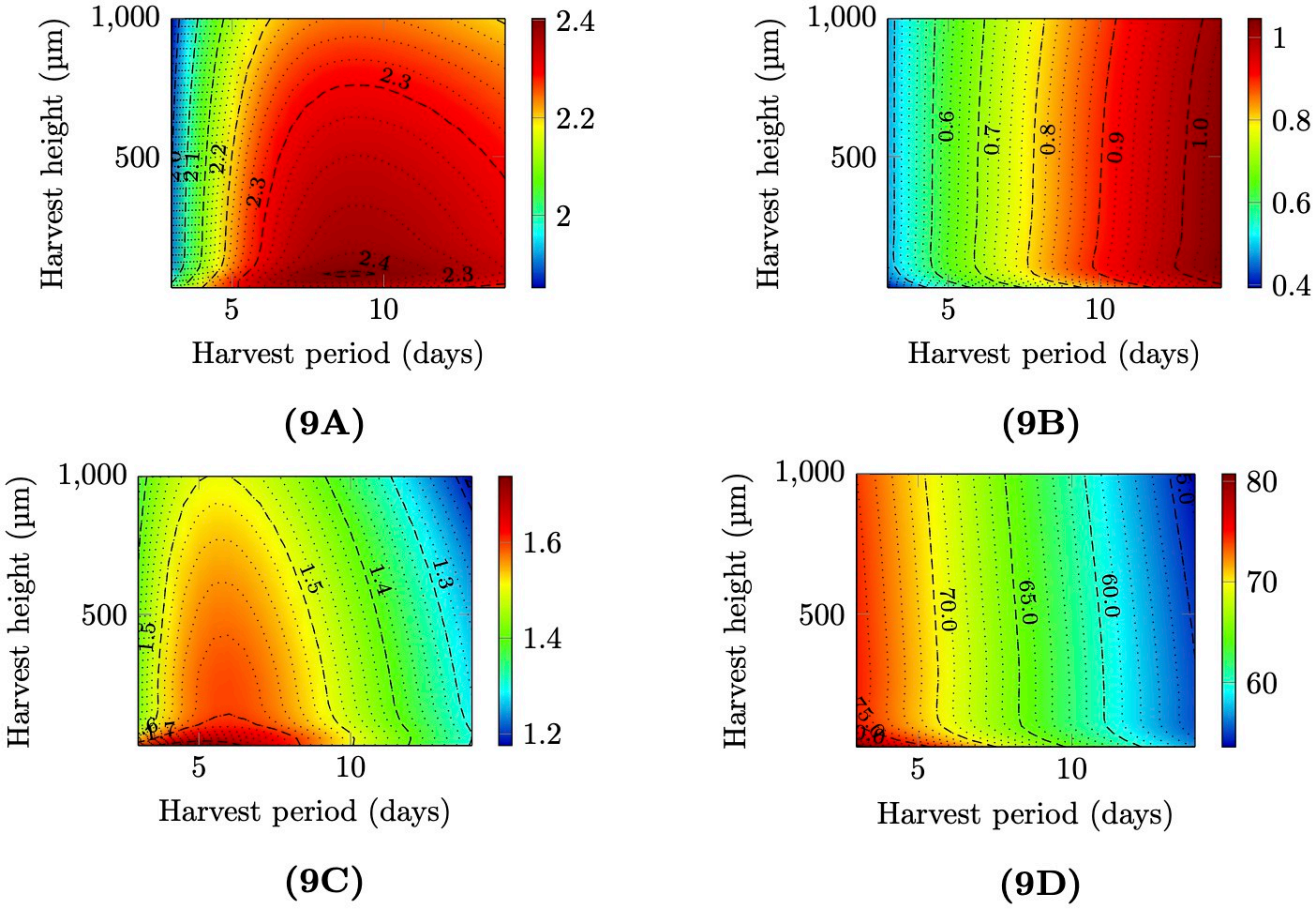
Daily production rates obtained using 1D numerical simulation (ie. uniform scraping pattern) for different biofilm components and biofilm compositions with respect to harvest period and height. Fig 9A: Daily production rate of dry biofilm (**A** + **N** + **E**). Fig 9B: Daily production rate of dry ECM (**E**). Fig 9C: Daily production rate of dry microalgae (**A** + **N**). Fig 9D: Biofilm composition: dry biomass percentage within microalgae (**A** + **N**)/(**A** + **N** + **E**).

At present, only few information are available regarding harvesting parameters [54, 55]. However, parameters such as period and the depth at which the biofilm is collected are important from a process point of view. For instance, the optimization of the harvesting frequency allows to maintain the biofilms in exponential growth (i.e. in optimal metabolic state) and once harvested the biofilm that is remaining on the support may even exhibit improved productivity (2 times higher) [9, 54, 56]. Extensive simulations with the 1D model are run to assess the impact of the remaining biofilm height after harvest and of the harvesting period. The harvest height is defined as the distance of the scrapping blade with respect to the support. For each couple of harvesting conditions, i.e. height and period, a one dimensional numerical simulation is run until the estimated daily production for each biofilm component between two harvests becomes lower than 0.1%. Fig 9 represents the daily production rate of dry biomass for each component (in *g*/*m*^2^/*d*) with respect to the different tested harvesting conditions. The simulation shows that the optimal harvesting period is between 7 and 8.4 days, which maximizes the average daily dry biofilm production. When combined to the optimal height of 75*μ*m for harvesting, a maximum productivity of 2.4 *g*/*m*^2^/*d* is reached. This value is in the lower range of several experimental works. In their outdoor experiments [57] record productivities in the range of 1.5 to 5 *g*/*m*^2^/*d*. Higher productivities can though be reached if the model accounts for the liquid flow at the surface of the biofilm. This would enhance the transfer rate from the liquid to the biofilm, and in particular reduce the oxygen accumulation in the biofilm which has a noticeable inhibiting effect on productivity. In industrial set-up, as in rotative photobioreactors [58], a supply of CO_2_ will enhance growth, and this option is not considered here. [54] found that an harvesting frequency of 7 days, similar to the one identified by our simulation, allowed to maximise productivity and nutrient removal from wastewater. However, the authors reported that a harvesting height of 2mm doubled productivity with respect to an height of 130 *μ*m [54]. A lower density and fluffy structure were reported for those thick biofilms. In our simulation, harvesting heights larger than 100*μ*m did not significantly affect productivity. It has to be pointed out that multi-species biofims were studied in [54]. Accordingly, harvesting height affected species dominance and in turn biofilm structure which may have boosted productivity. In our biofilm, a higher height after harvest means that a great fraction of the biofilm, would not be illuminated or reached by nutrients. Production of biomass in these layers will therefore decrease because of respiration and mortality, leading to an overall decrease of carbon fixed into biomass. Consequently, it results that there is a trade-off corresponding to the thickness of the active front.

This analysis must be refined when focusing on a specific biofilm component. If algal biomass is targeted, a more frequent harvest (every 5 days) at a thinner depth (20*μm*) is optimal. Fig 9D shows that the fraction of algal biomass in the biofilm mainly decreases with the harvesting period.

Different choices of harvesting heights and harvesting periods can lead to the same biofilm productivity. For example the iso-productivity at 2.3 *g*.*m*^−2^.*d*^−1^ encompasses a large parameter range. However, the composition of the biofilm can be very different, with more algae and less EPS if the biofilm is harvested often at a lower depth. The harvesting strategy must then be taylored to the desired production objectives.

### 3.6 Impact of scrapping pattern on productivity

**Fig 10:**
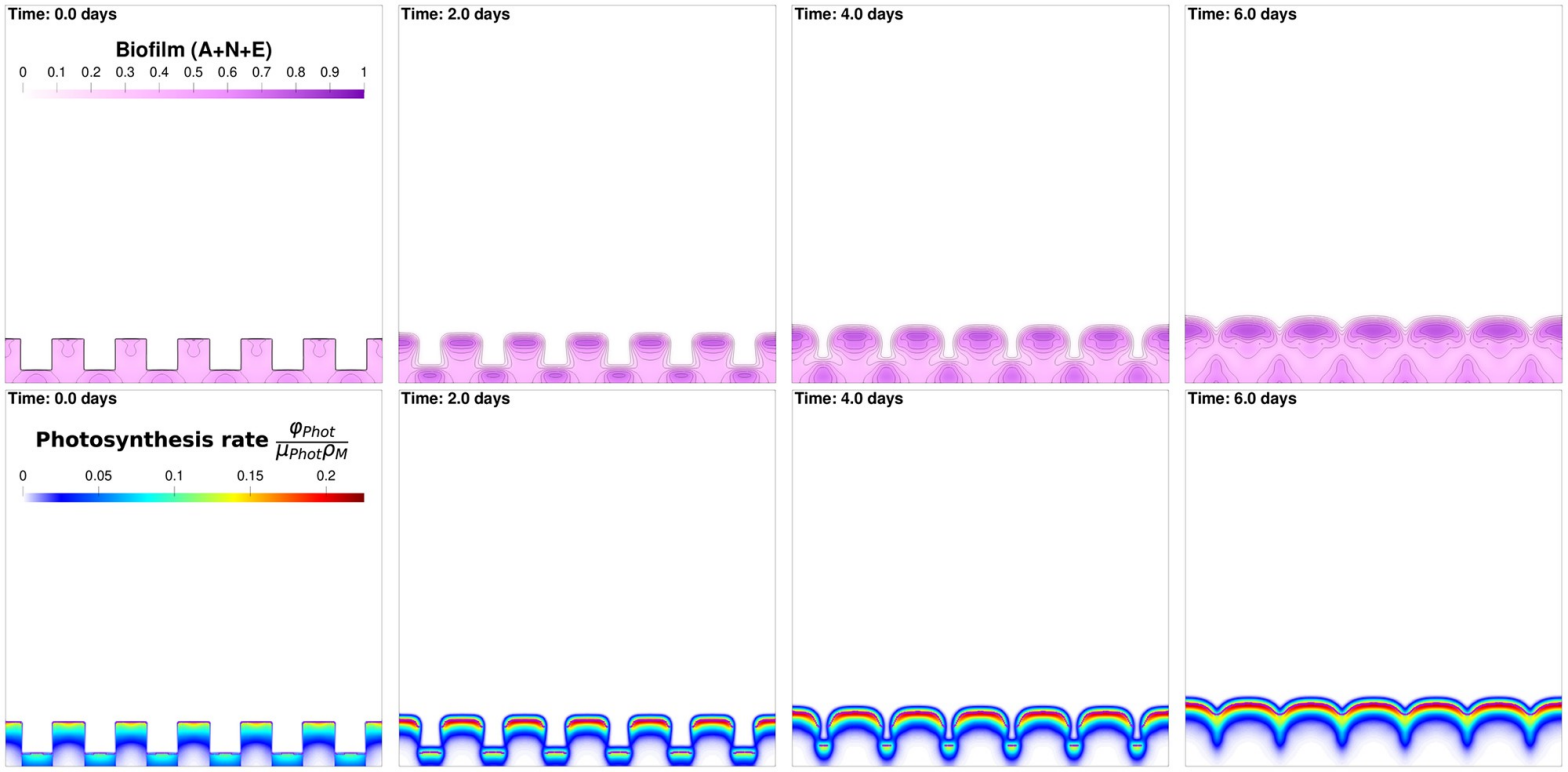
Biofilm (first row) and photosynthesis rate (second row) over an harvesting period. Harvest is performed every 6.5 days, using battlement scraping shape of width 250*μm*. The first column, at time t = 0, represents biofilm and photosynthesis rate immediately after the harvest and the last column 6 days after harvest (or equivalently half a day before the second harvest). The columns in between represent the intermediary times *t* = 2 and *t* = 4 days after harvest. All these subfigures correspond to square domain of length *L*_*x*_ = *L*_*z*_ = 3 · 10^−3^.

In this section, the 2D simulation model is used to determine how the scrapping pattern affects productivity and biofilm structure compared to flat harvesting. Different scrapping are simulated, assuming that they are all square-wave with different depths, see Fig 10 for the case of a harvest with a battlement scraping shape of size 250*μm* and a harvesting period of 6.5 days.

With a crenellated scrapping pattern, the expected productivity gain is in the range of 10% compared to the best strategies obtained with uniform harvesting (see Table 2) for optimising algal biomass *M*. The main effect of the tooth pattern is that it increases the contact surface between the biofilm and the liquid medium. Hence, the inner parts of the biofilm are better exposed to nutrients through diffusion. In the same way, the oxygen can better be released in the liquid bulk. Even if this effect is not represented, light which is highly diffused in this very turbid environment, can also reach the cells along the side of the hole created by the scraping tool.

**Table 2:** Productivity comparison for harvest using crenellated scraping shape of size 250*μm*, compared with uniform harvest at the lower/upper height of the crenels (with identical harvest period).

Period (days)	4			6.5			9		
Min height (*μm*)	100			100			75		
Max height (*μm*)	350			350			325		
Component	Cre.	Min	Max	Cre.	Min	Max	Cre.	Min	Max
A (g/m^2^/day)	1.427	1.271	1.256	1.423	1.294	1.259	1.318	1.350	1.330
M (g/m^2^/day)	1.686	1.500	1.483	1.687	1.548	1.505	1.576	1.600	1.576
E (g/m^2^/day)	0.685	0.538	0.539	0.833	0.854	0.862	0.944	0.732	0.729
B (g/m^2^/day)	2.371	2.039	2.022	2.521	2.403	2.367	2.520	2.332	2.305

It results that the crenel strategy is a way to harvest the same quantities as the optimal strategy (with 9 days and 75*μm*), but with a shorter period. It can be more adapted from an operative point of view. Typically, at industrial scale, it is more convenient to harvest every week at the same time, instead of harvesting every 9 days. The tooth pattern does not significantly improve the productivity compared to the best results obtained for the optimal flat harvesting conditions.

When harvesting at higher frequency (every 4 or 6.5 days), the gain can vary when considering a specific component in the biofilm. The productivity for the storage compound *A* is enhanced, while there is no strong gain when considering the whole biofilm. It is worth remarking that productivity with a crenel shape is always better than the productivity with a flat harvesting at the surface or at the bottom of the crenels (with the same harvesting frequency). The chosen crenel strategy is however detrimental to EPS productivity. A dedicated strategy must be explored if EPS is the goal of the process. These conclusions can be modulated after the model is upgraded to account for the liquid flow at the surface of the biofilm. It is likely that the crenel shape would lead to stronger exchanges of CO_2_ and O_2_ with lower oxygen inhibition.

However, considering the limited expected gains, the costs of implementing a complicated crenelated harvesting setup would probably not be compensated by the slight productivity increase.

## 4 Conclusion

We propose a 2D mixture model to simulate structural and metabolic dynamics in photosynthetic biofilms. The impact of various harvesting strategies on productivity was also assessed. The main originality of this model is the accurate description of the biological mechanisms involved in biofilm formation and development. The results show that our model predicts structures similar to those reported in experimental studies on photosynthetic biofilms. Also, simulations of light and nutrient profiles allowed to understand the already observed stratification of active cell layers with respect to the dead organic matter that seems to support the former ones. Interestingly, model results show that the harvesting strategy (frequency and pattern) must be optimised considering the product targeted.

Future challenges would be the integration of different cell phenotyphes (e.g. cells in different phases of cell cycle) or genotypes (i.e multi-species biofilms) to further investigate spatial patterns such as microbial vertical distribution in photosynthetic biofilms. From a more applied point of view, the model could be extended to more complicated reactor designs than a simple tank reactor. Indeed, several working groups experiment the idea of cultivating microalgae biofilms on rotating systems [59], where the biofilm is periodically submerged and illuminated. It would be of great interest to adapt the present model to this specific case and to optimize productivity by playing on factors, such as light frequency, light intensity or rotating frequency.

## Supporting information

S1 Text Physical model. (PDF)

S2 Text Complete system of PDEs. (PDF)

S3 Text Initial data and parameter values. (PDF)

S4 Text Numerical scheme. (PDF)

S5 Text Photosynthesis rate insight for multi-spot colony simulation. (PDF)

## References

[1] Wijffels RH, Barbosa MJ. An outlook on microalgal biofuels. Science. 2010;329(5993):796–799. doi: 10.1126/science.1189003 20705853

[2] Posten C, Schaub G. Microalgae and terrestrial biomass as source for fuels—a process view. Journal of biotechnology. 2009;142(1):64–69. doi: 10.1016/j.jbiotec.2009.03.015 19446353

[3] Rodolfi L, Chini Zittelli G, Bassi N, Padovani G, Biondi N, Bonini G, et al. Microalgae for oil: Strain selection, induction of lipid synthesis and outdoor mass cultivation in a low-cost photobioreactor. Biotechnology and bioengineering. 2009;102(1):100–112. doi: 10.1002/bit.22033 18683258

[4] Lardon L, Hélias A, Sialve B, Steyer JP, Bernard O. Life-Cycle Assessment of Biodiesel Production from Microalgae. Environmental Science & Technology. 2009;43(17):6475–6481. doi: 10.1021/es900705j 19764204

[5] Gross M, Wen Z. Yearlong evaluation of performance and durability of a pilot-scale Revolving Algal Biofilm (RAB) cultivation system. Bioresource Technology. 2014;171:50–58. doi: 10.1016/j.biortech.2014.08.052 25189508

[6] Kesaano M, Sims RC. Algal biofilm based technology for wastewater treatment. Algal Research. 2014;5:231–240. doi: 10.1016/j.algal.2014.02.003

[7] Gross M, Jarboe D, Wen Z. Biofilm-based algal cultivation systems. Applied microbiology and biotechnology. 2015;99(14):5781–5789. doi: 10.1007/s00253-015-6736-5 26078112

[8] Blanken W, Janssen M, Cuaresma M, Libor Z, Bhaiji T, Wijffels R. Biofilm growth of Chlorella sorokiniana in a rotating biological contactor based photobioreactor. Biotechnology and bioengineering. 2014;111(12):2436–2445. doi: 10.1002/bit.25301 24895246

[9] Christenson LB, Sims RC. Rotating algal biofilm reactor and spool harvester for wastewater treatment with biofuels by-products. Biotechnology and bioengineering. 2012;109(7):1674–1684. doi: 10.1002/bit.24451 22328283

[10] Liu T, Wang J, Hu Q, Cheng P, Ji B, Liu J, et al. Attached cultivation technology of microalgae for efficient biomass feedstock production. Bioresource technology. 2013;127:216–222. doi: 10.1016/j.biortech.2012.09.100 23131644

[11] Flemming HC, Wingender J. The biofilm matrix. Nature reviews microbiology. 2010;8(9):623–633. doi: 10.1038/nrmicro2415 20676145

[12] Bridier A, Meylheuc T, Briandet R. Realistic representation of Bacillus subtilis biofilms architecture using combined microscopy (CLSM, ESEM and FESEM). Micron. 2013;48:65–69. doi: 10.1016/j.micron.2013.02.013 23517761

[13] Wang J, Liu W, Liu T. Biofilm based attached cultivation technology for microalgal biorefineries—a review. Bioresource technology. 2017;244:1245–1253. doi: 10.1016/j.biortech.2017.05.136 28576483

[14] Bauer E, Zimmermann J, Baldini F, Thiele I, Kaleta C. BacArena: Individual-based metabolic modeling of heterogeneous microbes in complex communities. PLOS Computational Biology. 2017;13(5):1–22. doi: 10.1371/journal.pcbi.1005544 28531184 PMC5460873

[15] Clarelli F, Di Russo C, Natalini R, Ribot M. A fluid dynamics multidimensional model of biofilm growth: stability, influence of environment and sensitivity. Mathematical medicine and biology: a journal of the IMA. 2016;33(4):371–395. doi: 10.1093/imammb/dqv024 26188019

[16] van Loosdrecht MCM, Heijnen JJ, Eberl H, Kreft J, Picioreanu C. Mathematical modelling of biofilm structures. Antonie van Leeuwenhoek. 2002;81(1):245–256. doi: 10.1023/A:1020527020464 12448723

[17] Jayathilake PG, Gupta P, Li B, Madsen C, Oyebamiji O, González-Cabaleiro R, et al. A mechanistic Individual-based Model of microbial communities. PLOS ONE. 2017;12(8):1–26. doi: 10.1371/journal.pone.0181965 28771505 PMC5542553

[18] Li B, Taniguchi D, Gedara JP, Gogulancea V, Gonzalez-Cabaleiro R, Chen J, et al. NUFEB: A massively parallel simulator for individual-based modelling of microbial communities. PLOS Computational Biology. 2019;15(12):1–23. doi: 10.1371/journal.pcbi.1007125 31830032 PMC6932830

[19] Alpkvist E, Picioreanu C, van Loosdrecht MC, Heyden A. Three-dimensional biofilm model with individual cells and continuum EPS matrix. Biotechnology and bioengineering. 2006;94(5):961–979. doi: 10.1002/bit.20917 16615160

[20] Alpkvista E, Klapper I. A multidimensional multispecies continuum model for heterogeneous biofilm development. Bulletin of mathematical biology. 2007;69(2):765–789. doi: 10.1007/s11538-006-9168-7 17211734

[21] Truesdell C. Sulle basi della termomeccanica. I, II. Atti Accad Naz Lincei Rend Cl Sci Fis Mat Nat (8). 1957;22:33–38, 158–166.

[22] Clarelli F, Di Russo C, Natalini R, Ribot M. A fluid dynamics model of the growth of phototrophic biofilms. Journal of mathematical biology. 2013;66(7):1387–1408. doi: 10.1007/s00285-012-0538-5 22562622

[23] Klapper I, Dockery J. Finger formation in biofilm layers. SIAM Journal on Applied Mathematics. 2002;62(3):853–869. doi: 10.1137/S0036139900371709

[24] Zhang T, Cogan N, Wang Q. Phase-field models for biofilms II. 2-D numerical simulations of biofilm-flow interaction. Commun Comput Phys. 2008;4(1):72–101.

[25] Polizzi B, Bernard O, Ribot M. A time-space model for the growth of microalgae biofilms for biofuel production. Journal of Theoretical Biology. 2017;432:55–79. doi: 10.1016/j.jtbi.2017.08.017 28826969

[26] Xiao R, Zheng Y. Overview of microalgal extracellular polymeric substances (EPS) and their applications. Biotechnology Advances. 2016;34(7):1225–1244. doi: 10.1016/j.biotechadv.2016.08.004 27576096

[27] Bernard O, Boulanger AC, Bristeau MO, Sainte-Marie J. A 2D model for hydrodynamics and biology coupling applied to algae growth simulations. ESAIM: Mathematical Modelling and Numerical Analysis. 2013;47(5):1387–1412. doi: 10.1051/m2an/2013072

[28] Mairet F, Bernard O, Masci P, Lacour T, Sciandra A. Modelling neutral lipid production by the microalga Isochrysis aff. galbana under nitrogen limitation. Bioresource technology. 2011;102(1):142–149. doi: 10.1016/j.biortech.2010.06.138 20656476

[29] Eilers P, Peeters J. Dynamic behaviour of a model for photosynthesis and photoinhibition. Ecological modelling. 1993;69(1-2):113–133. doi: 10.1016/0304-3800(93)90052-T

[30] Costache T, Fernández FGA, Morales M, Fernández-Sevilla J, Stamatin I, Molina E. Comprehensive model of microalgae photosynthesis rate as a function of culture conditions in photobioreactors. Applied microbiology and biotechnology. 2013;97(17):7627–7637. doi: 10.1007/s00253-013-5035-2 23793345

[31] Staats N, Stal LJ, Mur LR. Exopolysaccharide production by the epipelic diatom Cylindrotheca closterium: effects of nutrient conditions. Journal of Experimental Marine Biology and Ecology. 2000;249(1):13–27. doi: 10.1016/S0022-0981(00)00166-0 10817825

[32] Peng L, Lan CQ, Zhang Z. Evolution, detrimental effects, and removal of oxygen in microalga cultures: a review. Environmental Progress & Sustainable Energy. 2013;32(4):982–988. doi: 10.1002/ep.11841

[33] Wilking JN, Angelini TE, Seminara A, Brenner MP, Weitz DA. Biofilms as complex fluids. MRS bulletin. 2011;36(5):385–391. doi: 10.1557/mrs.2011.71

[34] Blauert F, Horn H, Wagner M. Time-resolved biofilm deformation measurements using optical coherence tomography. Biotechnology and bioengineering. 2015;112(9):1893–1905. doi: 10.1002/bit.25590 25786671

[35] Truesdell C, Rajagopal KR. An introduction to the mechanics of fluids. Springer Science & Business Media; 2010.

[36] Cogan N, Keener JP. The role of the biofilm matrix in structural development. Mathematical medicine and biology: a journal of the IMA. 2004;21(2):147–166. doi: 10.1093/imammb/21.2.147 15228104

[37] Fanesi A, Lavayssière M, Breton C, Bernard O, Briandet R, Lopes F. Shear stress affects the architecture and cohesion of Chlorella vulgaris biofilms. Scientific Reports. 2021;11(1):1–11. doi: 10.1038/s41598-021-83523-3 33597585 PMC7889892

[38] Derlon N, Massé A, Escudié R, Bernet N, Paul E. Stratification in the cohesion of biofilms grown under various environmental conditions. Water research. 2008;42(8-9):2102–2110. doi: 10.1016/j.watres.2007.11.016 18086485

[39] Hwang G, Klein MI, Koo H. Analysis of the mechanical stability and surface detachment of mature Streptococcus mutans biofilms by applying a range of external shear forces. Biofouling. 2014;30(9):1079–1091. doi: 10.1080/08927014.2014.969249 25355611

[40] Bridier A, Bridier A, Dubois-Brissonnet F, Boubetra A, Thomas V, Briandet R. The biofilm architecture of sixty opportunistic pathogens deciphered using a high throughput CLSM method. Journal of microbiological methods. 2010;82(1):64–70. doi: 10.1016/j.mimet.2010.04.006 20433880

[41] Bridier A, Le Coq D, Dubois-Brissonnet F, Thomas V, Aymerich S, Briandet R. The Spatial Architecture of Bacillus subtilis Biofilms Deciphered Using a Surface-Associated Model and In Situ Imaging. PLOS ONE. 2011;6(1):1–10. doi: 10.1371/journal.pone.0016177 21267464 PMC3022735

[42] Stewart PS, Franklin MJ. Physiological heterogeneity in biofilms. Nature Reviews Microbiology. 2008;6(3):199–210. doi: 10.1038/nrmicro1838 18264116

[43] Lichtenberg M, Brodersen KE, Kühl M. Radiative energy budgets of phototrophic surface-associated microbial communities and their photosynthetic efficiency under diffuse and collimated light. Frontiers in microbiology. 2017;8:452. doi: 10.3389/fmicb.2017.00452 28400749 PMC5368174

[44] Pringault O, Garcia-Pichel F. Monitoring of oxygenic and anoxygenic photosynthesis in a unicyanobacterial biofilm, grown in benthic gradient chamber. FEMS microbiology ecology. 2000;33(3):251–258. doi: 10.1111/j.1574-6941.2000.tb00747.x 11098076

[45] Bernstein HC, Kesaano M, Moll K, Smith T, Gerlach R, Carlson RP, et al. Direct measurement and characterization of active photosynthesis zones inside wastewater remediating and biofuel producing microalgal biofilms. Bioresource technology. 2014;156:206–215. doi: 10.1016/j.biortech.2014.01.001 24508901

[46] Häubner N, Schumann R, Karsten U. Aeroterrestrial microalgae growing in biofilms on facades—response to temperature and water stress. Microbial ecology. 2006;51(3):285–293. doi: 10.1007/s00248-006-9016-1 16596441

[47] de Beer D, Meyer V, Klatt J, Li T. Photosynthesis under very high oxygen concentrations in dense microbial mats and biofilms. bioRxiv. 2018; p. 335299.

[48] Tresse O, Lescob S, Rho D. Dynamics of living and dead bacterial cells within a mixed-species biofilm during toluene degradation in a biotrickling filter. Journal of applied microbiology. 2003;94(5):849–854. doi: 10.1046/j.1365-2672.2003.01914.x 12694450

[49] Barnett A, Méléder V, Blommaert L, Lepetit B, Gaudin P, Vyverman W, et al. Growth form defines physiological photoprotective capacity in intertidal benthic diatoms. The ISME journal. 2015;9(1):32–45. doi: 10.1038/ismej.2014.105 25003964 PMC4274417

[50] Picioreanu C, Van Loosdrecht MC, Heijnen JJ. Mathematical modeling of biofilm structure with a hybrid differential-discrete cellular automaton approach. Biotechnology and bioengineering. 1998;58(1):101–116. doi: 10.1002/(SICI)1097-0290(19980405)58:1<101::AID-BIT11>3.0.CO;2-M 10099266

[51] Fanesi A, Paule A, Bernard O, Briandet R, Lopes F. The Architecture of Monospecific Microalgae Biofilms. Microorganisms. 2019;7(9). doi: 10.3390/microorganisms7090352 31540235 PMC6780892

[52] Paula AJ, Hwang G, Koo H. Dynamics of bacterial population growth in biofilms resemble spatial and structural aspects of urbanization. Nature communications. 2020;11(1):1–14. doi: 10.1038/s41467-020-15165-4 32170131 PMC7070081

[53] Nadell CD, Drescher K, Foster KR. Spatial structure, cooperation and competition in biofilms. Nature Reviews Microbiology. 2016;14(9):589–600. doi: 10.1038/nrmicro.2016.84 27452230

[54] Boelee N, Janssen M, Temmink H, Taparavičiūtė L, Khiewwijit R, Jánoska Á, et al. The effect of harvesting on biomass production and nutrient removal in phototrophic biofilm reactors for effluent polishing. Journal of applied phycology. 2014;26(3):1439–1452. doi: 10.1007/s10811-013-0178-1

[55] Choudhary P, Prajapati SK, Kumar P, Malik A, Pant KK. Development and performance evaluation of an algal biofilm reactor for treatment of multiple wastewaters and characterization of biomass for diverse applications. Bioresource technology. 2017;224:276–284. doi: 10.1016/j.biortech.2016.10.078 27818159

[56] Schnurr PJ, Allen DG. Factors affecting algae biofilm growth and lipid production: A review. Renewable and Sustainable Energy Reviews. 2015;52:418–429. doi: 10.1016/j.rser.2015.07.090

[57] Gross M, Henry W, Michael C, Wen Z. Development of a rotating algal biofilm growth system for attached microalgae growth with in situ biomass harvest. Bioresource technology. 2013;150:195–201. doi: 10.1016/j.biortech.2013.10.016 24161650

[58] Blanken W, Janssen M, Cuaresma M, Libor Z, Bhaiji T, Wijffels R. Biofilm growth of Chlorella sorokiniana in a rotating biological contactor based photobioreactor. Biotechnology and bioengineering. 2014;111(12):2436–2445. doi: 10.1002/bit.25301 24895246

[59] Morales M, Bonnefond H, Bernard O. Rotating algal biofilm versus planktonic cultivation: LCA perspective. Journal of Cleaner Production. 2020;257:120547. doi: 10.1016/j.jclepro.2020.120547

