# Supplementary material for: Understanding photosynthetic biofilm productivity and structure through 2D simulation
Source: PLoS Comput Biol. 2022 Apr 4;18(4):e1009904. doi: 10.1371/journal.pcbi.1009904 (PMC9037940; doi:10.1371/journal.pcbi.1009904)
Supplement: S1 Text — (PDF) [file pcbi.1009904.s001.pdf]

Understanding photosynthetic biofilm productivity and structure through 2D simulation

Bastien Polizzi[1*], Andrea Fanesi[2], Filipa Lopes[2], Magali Ribot[3], Olivier Bernard[4,5],

1 Laboratoire de Mathématiques de Besançon, Université Bourgogne Franche-Comté, CNRS UMR-6623, 16, route de Gray, 25030 Besançon Cedex, France
2 LGPM, CentraleSupélec, 3, rue Joliot-Curie 91192 Gif-Sur-Yvette Cedex, France
3 IDP, Université d'Orléans, CNRS, UMR CNRS 7013, rue de Chartres, BP 6759, F-45067 Orléans Cedex 2, France
4 BIOCORE, Inria Sophia Antipolis Méditerranée Research Centre, Valbonne, France
5 LOV-UPMC-CNRS, UMR 7093, Station Zoologique, Villefranche-sur-mer, France

Supporting information

S1 Physical model

The physical model is based on the mixture theory framework [1] which generalises Euler equations for multiphase fluid mechanics systems. Precisely, following [2], each component is described through its mass balance equation and its force balance equation. The model variables depend on time $t \in \mathbb{R}^+$ and space $X = \begin{pmatrix} x & z \end{pmatrix} \in [0, L_x] \times [0, L_z]$. Here the model is simulated in dimension 2, but the extension in this paper and the general equations proposed below are also valid in dimension 3. We present here the general form of the equations, all the details about the source terms and the reaction rates can be found in S2 Text.

The mass balance equations sum up all the considered mass fluxes between the compartments. The expressions of the source terms $(\Gamma_\phi)_{\phi \in \{A,N,E,L\}}$ for the mass balance equations are nonlinear functions reflecting the bioprocess rates. These nonlinear functions express how the biochemical constituents affect the bioreaction rates. The mass balance equations read

$$\partial_t \phi + \nabla_X \cdot (\phi \mathbf{v}_\phi) = \frac{\Gamma_\phi}{\rho_\phi} \tag{1}$$

where $\phi \in \{A, N, E, L\}$ is the component volume fraction, $\mathbf{v}_\phi$ is its transport velocity and ρ_ϕ its density. Since the mass balance equations are written in terms of volume fractions, the model includes a volume constraint, which says that the whole volume is occupied by the four constituents, that is to say

$$\sum_{\phi \in \{A,N,E,L\}} \phi = 1. \tag{2}$$

This constraint can be expressed equivalently as an incompressibility constraint, in the spirit of Navier-Stokes incompressibility constraint:

$$\sum_{\phi \in \{A,N,E,L\}} \left(\nabla_X \cdot (\phi \mathbf{v}_\phi) - \frac{\Gamma_\phi}{\rho_\phi} \right) = 0. \tag{3}$$

The force balance equations gather all the model physical features. So, as in [2], we only consider the hydrostatic pressure P, the elastic tensor $\gamma_\phi \mathbf{I}$, the friction between phases $\mathbf{m}_\phi$ and the momentum supply induced by mass exchanges $\Gamma_\phi \mathbf{v}_\phi$. Therefore, for $\phi \in \{M = A + N, E\}$, the generic form of the momentum balance equation writes

$$\rho_\phi \partial_t(\phi \mathbf{v}_\phi) + \rho_\phi \nabla_X \cdot (\phi \mathbf{v}_\phi \otimes \mathbf{v}_\phi) = -\phi \nabla_X P - \nabla_X \cdot (\gamma_\phi \mathbf{I} \phi) + \mathbf{m}_\phi + \Gamma_\phi \mathbf{v}_\phi, \quad (4)$$

with $\mathbf{I}$ the identity matrix. The momentum equation for the liquid phase slightly differs from this generic form. Indeed, the liquid is not elastic (ie. $\gamma_L = 0$) and in a closed system the total momentum supply is conserved thus the momentum supply for the liquid phase rewrites in terms of the momentum supply for the other phases, ie. $\mathbf{m}_L + \Gamma_L \mathbf{v}_L = -\sum_{\phi \neq L} (\mathbf{m}_\phi + \Gamma_\phi \mathbf{v}_\phi)$.

Inorganic carbon **C**, oxygen **O** and nitrate **S** are dissolved components. They diffuse and are advected by the liquid phase. So, we do not write any momentum equation for these components and their mass balance equation takes the following form :

$$\partial_t(\theta L) + \nabla_X \cdot (\theta L \mathbf{v}_L) - \nabla_X \cdot (D_\theta L \nabla_X \theta) = \frac{\Gamma_\theta}{\rho_L}, \text{ for } \theta \in \{S, C, O\}, \quad (5)$$

with D_θ the diffusion coefficient.

To sum up, the general form of this model is the same as the model in [2], but it differs on several points. First, the physiology in the mass balance equations is described with many more details, which complicates the incompressibility constraint. Then, we consider three distinct velocities in the force balance equations : $\mathbf{v}_M$ for the microalgae, $\mathbf{v}_E$ for the extra-cellular matrix and $\mathbf{v}_L$ for the liquid phase. Finally, we couple the mixture model with complementary mass balance equations detailing the evolution of the components dissolved in liquid. The numerical scheme used to discretize the full system of equations is explained in S4 Text.

References

1. Truesdell C and Rajagopal KR. An introduction to the mechanics of fluids. Springer Science & Business Media, 2010.
2. Clarelli F, Di Russo C, Natalini R, and Ribot M. A fluid dynamics model of the growth of phototrophic biofilms. Journal of mathematical biology 2013;66:1387–408.
