# Supplementary material for: Understanding photosynthetic biofilm productivity and structure through 2D simulation
Source: PLoS Comput Biol. 2022 Apr 4;18(4):e1009904. doi: 10.1371/journal.pcbi.1009904 (PMC9037940; doi:10.1371/journal.pcbi.1009904)
Supplement: S2 Text — (PDF) [file pcbi.1009904.s002.pdf]

Understanding photosynthetic biofilm productivity and structure through 2D simulation

Bastien Polizzi[1*], Andrea Fanesi[2], Filipa Lopes[2], Magali Ribot[3], Olivier Bernard[4,5],

1 Laboratoire de Mathématiques de Besançon, Université Bourgogne Franche-Comté, CNRS UMR-6623, 16, route de Gray, 25030 Besançon Cedex, France
2 LGPM, CentraleSupélec, 3, rue Joliot-Curie 91192 Gif-Sur-Yvette Cedex, France
3 IDP, Université d'Orléans, CNRS, UMR CNRS 7013, rue de Chartres, BP 6759, F-45067 Orléans Cedex 2, France
4 BIOCORE, Inria Sophia Antipolis Méditerranée Research Centre, Valbonne, France
5 LOV-UPMC-CNRS, UMR 7093, Station Zoologique, Villefranche-sur-mer, France

Supporting information

S2 Complete system of PDEs

This appendix summarises the full set of equations already mentioned in S1 Text, including a precise expression of all the terms. We already derived this model in [1]; here, we include some modifications for the inorganic carbon and oxygen supply, see eqs. (6) and (7).

We first recall for clarity the unknowns of the system, which depend on time and space. They are all defined on space domain $\Omega = [0, L_x] \times [0, L_z]$ of width L_x and height L_z.

Volume and mass fractions A (resp. N, E, L) stands for the carbon storage (resp. functional biomass, extra-cellular matrix, liquid) volume fraction; C (resp. O, S) stands for the inorganic carbon (resp. oxygen, nitrate) mass fraction in liquid.

Velocities $\mathbf{v}_M$ (resp. $\mathbf{v}_E$, $\mathbf{v}_L$) denotes the microalgae (resp. EPS, liquid) velocity.

We now describe the various reaction rates, that are limited by several factors.

Reaction rates We define first the light intensity I that is received by the biofilm at time t and depth z, see [1], the intra-cellular quota of functional biomass Q, the ratio $\hat{O}$ (resp. $\hat{I}$) between oxygen (resp. light intensity) concentration and optimal oxygen $\mathcal{K}_D$ (resp. optimal light intensity I_{opt}) concentration , namely :

$$I(t,z) = I_0(t)\exp\left(-\int_z^{L_z} \tau_L L(t,\xi) + \tau_M(A(t,\xi)+N(t,\xi)+E(t,\xi))d\xi\right),$$
$$Q = N/(N+A), \qquad \hat{O} = O/\mathcal{K}_D, \qquad \hat{I} = I/I_{opt},$$

where τ_M (resp. τ_L) is the absorption coefficient for the solid (resp. liquid) phase, see Table C in S3 Text. We now detail the expression of the various factors that limit the photosynthesis rate, that is to say light (resp. intra-cellular quota, liquid,

CO_2 and oxygen) limiting factor f_{Light} (resp. f_{Droop}, f_{Liquid}, $f_{[CO_2]}$, f_{Oxy}) as

$$f_{Light} = \frac{2(1+\mathcal{K}_I)\hat{I}}{\hat{I}^2 + 2\mathcal{K}_I\hat{I} + 1} \qquad f_{Droop} = \frac{Q_{max}}{Q_{max} - Q_{min}} \max\left\{0, 1 - \frac{Q_{min}}{\min\{Q, Q_{max}\}}\right\}$$
$$f_{Liquid} = \frac{(\mathcal{K}_L + 1)L}{\mathcal{K}_L + L} \qquad f_{[CO_2]} = \frac{C}{\mathcal{K}_C + C} \qquad f_{Oxy} = \frac{1}{1 + \left(\frac{O}{\mathcal{K}_O}\right)^{\alpha}};$$

parameters $\mathcal{K}_I$, $\mathcal{K}_L$, $\mathcal{K}_C$, $\mathcal{K}_O$, α, Q_{min} and Q_{max} are all estimated and defined at Table C in S3 Text.

The photosynthesis rate is therefore the product of these limiting factors; we also give the expression of the reaction rates for respiration, functional biomass production, EPS excretion (from N and from A) and death (from N and from A) with limiting factors depending on oxygen, nitrate and intra-cellular quota Q, as follows :

$$\begin{aligned}
\varphi_{Phot} &= \mu_{Phot}\rho_M N f_{[CO2]} f_{Liquid} f_{Oxy} f_{Droop} f_{Light}, \\
\varphi_{Resp} &= \mu_{Resp}\rho_M A \frac{O}{\mathcal{K}_R + O}, \\
\varphi_{Func} &= \mu_{Func}\rho_M N \frac{S}{\mathcal{K}_S + S} \max\left\{0, \frac{Q_{max} - \max\{Q, Q_{min}\}}{Q_{max} - Q_{min}}\right\}, \\
\varphi^A_{Excr} &= \mu^A_{Excr}\rho_M A \max\left\{0, \frac{Q_{max} - \max\{Q, Q_{min}\}}{Q_{max} - Q_{min}}\right\}, \\
\varphi^N_{Excr} &= \mu^N_{Excr}\rho_M N \max\left\{0, \frac{Q_{max} - \max\{Q, Q_{min}\}}{Q_{max} - Q_{min}}\right\}, \\
\varphi^A_{Death} &= \mu_{Death}\rho_M A \left(1 - \frac{\beta\hat{O}}{\hat{O}^\beta + \beta - 1}\right), \\
\varphi^N_{Death} &= \mu_{Death}\rho_M N \left(1 - \frac{\beta\hat{O}}{\hat{O}^\beta + \beta - 1}\right);
\end{aligned}$$

ρ_M stands for the microalgae density and the maximum reaction rate μ_{Phot} (resp.μ_{Resp}, μ_{Func}, μ^A_{Excr}, μ^N_{Excr}, μ_{Death}) for photosynthesis (resp. respiration, functional biomass production, EPS excretion from N, EPS excretion from A and death) is estimated and described at Table A in S3 Text and parameters $\mathcal{K}_R$, $\mathcal{K}_S$ and β, at Table C in S3 Text.

Dissolved inorganic carbon supply Finally, we describe more precisely dissolved inorganic carbon supply and oxygen supply, since it is a novelty of this paper with respect to [1]. We first give the expression for the oxygen supply rate φ^O_{Henry} and the dissolved inorganic carbon supply rate φ^C_{Henry}, defined thanks to Henry's law :

$$\varphi^O_{Henry} = -k^O_{L,a}(O - O_{eq})\mathbb{1}_{L>L_{lim}}, \tag{6}$$
$$\varphi^C_{Henry} = -m_C k^C_{L,a}([CO_2] - K_{\mathcal{H}} P_{CO_2})\mathbb{1}_{L>L_{lim}}, \tag{7}$$

where

$$[CO_2] = \frac{Alk}{K_1} \cdot \frac{h^2}{h + 2K_2}$$

with

$$h(r) = \left(-1 + r + \sqrt{(1 - 2r)\left(1 - \frac{4K_2}{K_1}\right) + r^2}\right)\frac{K_1}{2} \tag{8}$$

and

$$r = \max\left\{\frac{1}{2}, \frac{LC\rho_L}{Alk \cdot m_C}\right\}.$$

Here $k^O_{L,a}$ (resp. $k^C_{L,a}$) is the gas-liquid transfer rate for oxygen (resp. for dissolved inorganic carbon), O_{eq} is the oxygen equilibrium concentration in water, L_{lim} is a threshold value for the liquid volume fraction, m_C the inorganic molar mass, $K_{\mathcal{H}}$ Henry's constant for carbon dioxide, P_{CO_2} the partial pressure of carbon dioxide in the gaseous phase, Alk the alkalinity and K_1 and K_2 the dissociation constants for bicarbonate and carbonate respectively. The parameter values can be found in [2] and are given at Table E in S3 Text.

Now let us explain how we obtained these equations : the Henry's law for oxygen and dissolved inorganic carbon supply (in mol/L/d) can be expressed as

$$\tilde{\varphi}^C_{Henry} = -k^C_{L,a}\big([CO_2] - K_{\mathcal{H}} P_{CO_2}\big)$$
$$\tilde{\varphi}^O_{Henry} = -k^O_{L,a}(O - O_{eq}),$$

Since the gas-liquid exchanges take place in the bulk liquid, a threshold effect with respect to the liquid volume fraction is included, using the indicator function $\mathbb{1}_{L>L_{lim}}$. Now, following [2], the carbon dioxide concentration in the liquid $[CO_2]$ is given by

$$[CO_2] = \frac{Alk}{K_1} \cdot \frac{h^2}{h + 2K_2};$$

h is the proton concentration (ie. $[H^+]$), which can be estimated from formula

$$h(r) = \left(-1 + r + \sqrt{(1-2r)\left(1 - \frac{4K_2}{K_1}\right) + r^2}\right)\frac{K_1}{2}, \quad \text{with} \quad r = \max\left\{\frac{1}{2}, \frac{LC\rho_L}{Alk \cdot m_C}\right\}$$

where m_C is the molar mass of the inorganic carbon C. Since the dissolved inorganic carbon is mostly made of carbonate, we take $m_C = m_{CO_3^{2-}}$.

Source terms From the description of the reaction rates, we can easily deduce the source terms of Eq.(1)-(5), see [1] for more details. We obtain :

$$\Gamma_A = \varphi_{Phot} - \varphi_{Resp} - \eta^A_{Func}\varphi_{Func} - \varphi^A_{Excr} - \varphi^A_{Death},$$
$$\Gamma_N = \varphi_{Func} - \varphi^N_{Excr} - \varphi^N_{Death},$$
$$\Gamma_E = \varphi^A_{Excr} + \varphi^N_{Excr} + \varphi^A_{Death} + \varphi^N_{Death},$$
$$\Gamma_L = \eta^L_{Resp}\varphi_{Resp} - \eta^L_{Phot}\varphi_{Phot},$$
$$\Gamma_C = \eta^C_{Resp}\varphi_{Resp} - \eta^C_{Phot}\varphi_{Phot} + \varphi^C_{Henry},$$
$$\Gamma_S = -\eta^S_{Func}\varphi_{Func}, \qquad \Gamma_O = \eta^O_{Phot}\varphi_{Phot} - \eta^O_{Resp}\varphi_{Resp} + \varphi^O_{Henry},$$

where $\eta^A_{Func}, \eta^L_{Resp}, \eta^L_{Phot}, \eta^C_{Resp}, \eta^C_{Phot}, \eta^S_{Func}, \eta^O_{Phot}, \eta^O_{Resp}$ are pseudo stoichiometric coefficients given at Table B in S3 Text.

For the sake of completeness, we rewrite mass balance equations (1) for carbon storage, functional biomass and extra-cellular matrix

Mass balance equations

$$\partial_t A + \nabla_X \cdot (A\mathbf{v}_M) = \Gamma_A/\rho_M,$$
$$\partial_t N + \nabla_X \cdot (N\mathbf{v}_M) = \Gamma_N/\rho_M,$$
$$\partial_t E + \nabla_X \cdot (E\mathbf{v}_E) = \Gamma_E/\rho_E,$$

where ρ_E denotes the EPS density, and mass balance equations (5) for inorganic carbon, oxygen, nitrate :

Mass balance equations for dissolved components

$$
\begin{aligned}
\partial_t(SL) + \nabla_X \cdot (SL\mathbf{v}_L) - \nabla_X \cdot (D_S L \nabla_X S) &= \frac{\Gamma_S}{\rho_L}, \\
\partial_t(CL) + \nabla_X \cdot (CL\mathbf{v}_L) - \nabla_X \cdot (D_C L \nabla_X C) &= \frac{\Gamma_C}{\rho_L}, \\
\partial_t(OL) + \nabla_X \cdot (OL\mathbf{v}_L) - \nabla_X \cdot (D_O L \nabla_X O) &= \frac{\Gamma_O}{\rho_L},
\end{aligned}
$$

where ρ_L denotes the liquid density and D_S (resp. D_C, D_O) the nitrate (resp. inorganic carbon, oxygen) diffusion coefficient, see Table D in S3 Text.

We also recall the volume constraint (2) and its equivalent formulation under the form of an incompressibility equation (3) :

Volume constraint and incompressibility constraint

$$
\begin{aligned}
&A + N + E + L = 1, \\
&\nabla_X \cdot ((A+N)\mathbf{v}_M + E\mathbf{v}_E + L\mathbf{v}_L) = \frac{\Gamma_A + \Gamma_N}{\rho_M} + \frac{\Gamma_E}{\rho_E} + \frac{\Gamma_L}{\rho_L}
\end{aligned}
$$

and the force balance equation (4) for microalgae, EPS and liquid :

Force balance equations

$$
\begin{aligned}
&\partial_t((A+N)\mathbf{v}_M) + \nabla_X \cdot ((A+N)\mathbf{v}_M \otimes \mathbf{v}_M) = \frac{1}{\rho_M}\Big(-(A+N)\nabla_X P - \gamma_M \nabla_X (A+N) \\
&\quad - m_{ML}(\mathbf{v}_M - \mathbf{v}_L) - m_{ME}(\mathbf{v}_M - \mathbf{v}_E) + (\Gamma_A + \Gamma_N)\mathbf{v}_M\Big), \\
&\partial_t(E\mathbf{v}_E) + \nabla_X \cdot (E\mathbf{v}_E \otimes \mathbf{v}_E) = \frac{1}{\rho_E}\Big(-E\nabla_X P - \gamma_E \nabla_X E \\
&\quad - m_{EL}(\mathbf{v}_E - \mathbf{v}_L) + m_{ME}(\mathbf{v}_M - \mathbf{v}_E) + \Gamma_E \mathbf{v}_E\Big), \\
&\partial_t(L\mathbf{v}_L) + \nabla_X \cdot (L\mathbf{v}_L \otimes \mathbf{v}_L) = \frac{1}{\rho_L}\Big(-L\nabla_X P + m_{ML}(\mathbf{v}_M - \mathbf{v}_L) + m_{EL}(\mathbf{v}_E - \mathbf{v}_L) \\
&\quad - (\Gamma_A + \Gamma_N)\mathbf{v}_M - \Gamma_E \mathbf{v}_E\Big),
\end{aligned}
$$

where γ_M (resp. γ_E) is the microalgae (resp. EPS) elastic tensor coefficient and m_{ML} (resp. m_{ME}, m_{EL}) the friction coefficient between microalgae and liquid (resp. microalgae and EPS, EPS and liquid), see Table F in S3 Text. The unknown P is the hydrostatic pressure and will be determined thanks to the incompressibility constraint.

Boundary conditions Now, to complete the system, we determine the boundary conditions. We split the boundary of Ω, denoted by $\partial\Omega$, in two distinct parts : $\{(x, z = L_z), x \in [0, L_x]\}$ and $\Gamma_{\neq L_z} = \partial\Omega \setminus \{(x, z = L_z), x \in [0, L_x]\}$.

We impose no flux boundary conditions on the whole domain, except on the boundary $z = L_z$, where an inflow of nitrate takes place and a velocity is imposed

in order to satisfy the incompressibility constraint. Boundary conditions therefore write as :

$$
\begin{aligned}
&\nabla_X \phi(t,X)\cdot\vec{n}\,|_{\partial\Omega}=0, && \phi = A,N,E,L,\\
&\mathbf{v}_\phi(t,X)\cdot\vec{n}\,|_{\Gamma_{\neq L_z}}=0, && \phi = M,E,L,\\
&\mathbf{v}_\phi(t,x,L_z)=\int_0^{L_z}\left(\frac{\Gamma_A+\Gamma_N}{\rho_M}+\frac{\Gamma_E}{\rho_E}+\frac{\Gamma_L}{\rho_L}\right)d\xi, && \phi = M,E,L,\\
&\nabla_X(SL)(t,X)\cdot\vec{n}\,|_{\Gamma_{\neq L_z}}=0, \qquad (SL)(t,x,L_z)=\theta_S, &&\\
&\nabla_X(\phi L)(t,X)\cdot\vec{n}\,|_{\partial\Omega}=0 && \phi = C,O,
\end{aligned}
$$

where θ_S is the external supply of substrate.

In the next section, we complement the equations with the definition of the initial data we use in this article and with the complete set of parameter values.

References

1. Polizzi B, Bernard O, and Ribot M. A time-space model for the growth of microalgae biofilms for biofuel production. Journal of Theoretical Biology 2017;432:55 –79.
2. Bernard O, Sciandra A, and Madani S. Multimodel analysis of the response of the coccolithophore Emiliania huxleyi to an elevation of pCO2 under nitrate limitation. Ecological Modelling 2008;211:324 –338.
