# Supplementary material for: Understanding photosynthetic biofilm productivity and structure through 2D simulation
Source: PLoS Comput Biol. 2022 Apr 4;18(4):e1009904. doi: 10.1371/journal.pcbi.1009904 (PMC9037940; doi:10.1371/journal.pcbi.1009904)
Supplement: S3 Text — (PDF) [file pcbi.1009904.s003.pdf]

Understanding photosynthetic biofilm productivity and structure through 2D simulation

Bastien Polizzi[1*], Andrea Fanesi[2], Filipa Lopes[2], Magali Ribot[3], Olivier Bernard[4,5],

1 Laboratoire de Mathématiques de Besançon, Université Bourgogne Franche-Comté, CNRS UMR-6623, 16, route de Gray, 25030 Besançon Cedex, France
2 LGPM, CentraleSupélec, 3, rue Joliot-Curie 91192 Gif-Sur-Yvette Cedex, France
3 IDP, Université d'Orléans, CNRS, UMR CNRS 7013, rue de Chartres, BP 6759, F-45067 Orléans Cedex 2, France
4 BIOCORE, Inria Sophia Antipolis Méditerranée Research Centre, Valbonne, France
5 LOV-UPMC-CNRS, UMR 7093, Station Zoologique, Villefranche-sur-mer, France

Supporting information

S3 Initial data and parameter values

S3.1 Initial data

Initial distributions of nitrate S_0, oxygen O_0 and inorganic carbon C_0 are set on the whole domain Ω with values

$$S_0 = \theta_S, \qquad C_0 = 1.62 \cdot 10^{-4}, \qquad O_0 = O_{eq},$$

where θ_S is given at Table C in S3 Text, O_{eq} at Table E in S3 Text and the value for C_0 is explained later on in this subsection.

Microalgae are only present initially in a part $\mathscr{D}$ of the whole domain, let $\mathbb{1}_{\mathscr{D}}$ be the characteristic function of this initial domain $\mathscr{D}$, which will be described thereafter. No EPS is present initially and liquid fills all the remaining part. We take therefore the initial condition as

$$A_0 = 6 \cdot 10^{-2} \mathbb{1}_{\mathscr{D}}, \quad N_0 = 1.0057 \cdot 10^{-2} \mathbb{1}_{\mathscr{D}}, \quad E_0 = 0, \quad L_0 = 1 - A_0 - N_0 - E_0$$

Three different domains are considered as initial domains for microalgae:

Uniform distribution: $\mathscr{D} = \left\{(x,z),\ x \in [0, L_x],\ 0 \leqslant z < L_z \times 5 \cdot 10^{-3}\right\}$

Single-spot colony:
$\mathscr{D} = \left\{(x,z),\ 0.45L_x \leqslant x \leqslant 0.55L_x,\ 0 \leqslant z \leqslant \frac{L_z}{10} \sin\left(\frac{\pi(x-0.45L_x)}{0.1L_x}\right)\right\}$

Multi-spot colony: $\mathscr{D} = \left\{(x,z),\ \frac{L_x}{6} \leqslant x \leqslant \frac{L_x}{6},\ 0 \leqslant z \leqslant \psi(x)\right\}$ with

$$\psi(x) = L_z e^{-\left(\frac{x-L_x/2}{0.35L_x}\right)^2} \left(0.3 \sin\left(\frac{\pi(x-L_x/6)}{2L_x/3}\right) - 0.1\right).$$

The initial condition for the inorganic carbon mass fraction C_0 is determined thanks to Henry's law (7) in order that φ^C_{Henry} vanishes initially; this leads to $[CO_2]_0 = K_{\mathcal{H}} P_{CO_2}$ at time $t = 0$.

Using equation (8) and the positivity of the proton concentration, we obtain the initial proton concentration

$$h_0 = \frac{[CO_2]_0 + \sqrt{[CO_2]_0^2 + \frac{8AlkK_2}{K_1}[CO_2]_0}}{2Alk} K_1.$$

Then using the chemical equilibrium relations between carbon dioxide, carbonate and bicarbonate, see [1] for details, initial condition for C reads as :

$$C_0 = \frac{m_C}{\rho_L}\left(1 + \frac{K_1}{h_0}\left(1 + \frac{K_2}{h_0}\right)\right)[CO_2]_0$$

S3.2 Parameter values

In this subsection, we detail the values of the various parameters of the system, namely :

- maximum reaction rates at Table A,
- pseudo stoichiometric coefficients at Table B,
- parameters involved in reaction rates at Table C,
- diffusion coefficients at Table D,
- parameters for Henry's laws (6)-(7) at Table E,
- friction coefficients and tensor coefficients at Table F.

Name	Value in d^{-1}	Mechanisms	Ref.
μ_{Phot}	2	Photosynthesis	[2,3]
μ_{Resp}	0.2	Respiration	[4]
μ_{Func}	10.64	Functional biomass synthesis	[5]
μ^A_{Excr}	0.3	EPS excretion from **A**	[6]
μ^N_{Excr}	0.1	EPS excretion from **N**	S3.3 Text
μ_{Death}	0.2	Microalgae death rate	[7]

S3 Table A. Estimated values for the maximum reaction rates.

S3.3 Estimation of the EPS excretion rates

According to [20], Table 3 microalgae EPS contains between 1% and 16.9% of proteins. Denoting $\pi_\%$ the percentage of proteins in the EPS, we have that $\max \varphi^N_{Excr} = \pi_\% \max \varphi^A_{Excr}$. Then, using the definition of the internal functional biomass quota Q, we deduce that

$$\mu^N_{Excr} = \pi \mu^A_{Excr} \frac{Q_{max}}{1 - Q_{max}}, \quad (9)$$

which leads to $\mu^N_{Excr} \in [0.016, 0.27]$.

Name	Value	Unit	Mechanisms	Component	
η^{A}_{Phot}	1	kg**A**/kg**A**	Photosynthesis	Pool of carbon storage	**A**
η^{O}_{Phot}	0.106	kg**O**/kg**A**		Oxygen	**O**
η^{C}_{Phot}	0.146	kg**C**/kg**A**		Carbon dioxide	**C**
η^{L}_{Phot}	0.960	kg**L**/kg**A**		Liquid	**L**
η^{A}_{Resp}	1	kg**A**/kg**A**	Respiration	Pool of carbon storage	**A**
η^{O}_{Resp}	0.106	kg**O**/kg**A**		Oxygen	**O**
η^{C}_{Resp}	0.146	kg**C**/kg**A**		Carbon dioxide	**C**
η^{L}_{Resp}	0.960	kg**L**/kg**A**		Liquid	**L**
η^{A}_{Func}	1.583	kg**A**/kg**L**	Functional biomass synthesis	Pool of carbon storage	**A**
η^{S}_{Func}	0.867	kg**S**/kg**L**		Substrate	**S**

S3 Table B. Estimated values of the pseudo stoichiometric coefficients.

Name	Value	Unit	Mechanism	Interpretation	Ref.
Q_{min}	$5.82 \cdot 10^{-2}$	-	Droop law	Functional biomass quota: Minimal threshold	[5]
Q_{max}	$1.57 \cdot 10^{-1}$	-		Functional biomass quota: Maximal threshold	[5]
$\mathcal{K}_I$	0.1	-	Light	Haldane law parameter	[8,9]
I_{opt}	$100 \cdot 10^{-6}$	$\mathrm{mol\,m^{-2}\,s^{-1}}$		Optimal light intensity	[8,9]
τ_L	0.1	$\mathrm{m^{-1}}$		Absorption coefficient for **L**	[8–10]
τ_M	$2.5 \cdot 10^4$	$\mathrm{m^{-1}}$		Absorption coefficient for **M**	[8,10,11]
$\mathcal{K}_O$	$3.2 \cdot 10^{-5}$	kg**O**/kg**L**	Photosynthesis	Oxygen: half saturation constant	[12]
α	14	-		Oxygen: sigmoid stiffness	[12]
$\mathcal{K}_L$	0.5	-		Liquid: half saturation constant	[13]
$\mathcal{K}_C$	$4.4 \cdot 10^{-6}$	kg**C**/kg**L**		Inorganic carbon: half saturation constant	[14]
$\mathcal{K}_R$	$1 \cdot 10^{-6}$	kg**O**/kg**L**	Respiration	Oxygen: half saturation constant	[15]
$\mathcal{K}_S$	$6.2 \cdot 10^{-8}$	kg**L**/kg**L**	Functional biomass synthesis	Substrate: half saturation constant	[2]
$\mathcal{K}_D$	$7.2 \cdot 10^{-6}$	kg**O**/kg**L**	Death	Oxygen: half saturation constant	
β	1.8352	-		Oxygen: sigmoid stiffness	
θ_S	$4 \cdot 10^{-5}$	kg**S**/kg**L**	Substrate intake	Boundary condition	[2,5]

S3 Table C. Estimated values of parameters involved in reaction rates.

Name	Value	Unit	Interpretation	Ref.
D_S	$1.47 \cdot 10^{-4}$	$\mathrm{m^2\,d^{-1}}$	Substrate diffusion coefficient	[16]
D_C	$1.80 \cdot 10^{-4}$	$\mathrm{m^2\,d^{-1}}$	Inorganic carbon diffusion coefficient	[17]
D_O	$1.98 \cdot 10^{-4}$	$\mathrm{m^2\,d^{-1}}$	Oxygen diffusion coefficient	[16]

S3 Table D. Estimated values of the diffusion coefficients

Name	Value	Unit	Ref.
L_{lim}	0.99		
$k_{L,a}^{C}$	93	1/day	[18]
$K_{\mathcal{H}}$	36.7	$mmol_{CO_2} L^{-1} \mu atm$	[1]
P_{CO_2}	545	μatm	[1]
Alk	2.25e-3	mol/L	[1]
K_1	1.382e-9	mol/L	[1]
K_2	1.189e-12	mol/L	[1]
m_C	60.01	g/mol	CO_3^{2-}
$k_{L,a}^{O}$	100	1/day	[18]
O_{eq}	$7.2 \cdot 10^{-6}$	$kg\boldsymbol{O}/kg\boldsymbol{L}$	[19]

S3 Table E. Parameter estimates for Henry's law used to describe gas-liquid exchanges for dissolved inorganic carbone and oxygen

Name	Value	Unit	Mechanism	Component(s)	Ref.
m_{ML}	20	$\mathrm{kg\,m^{-3}\,d^{-1}}$	Friction coefficient	**M** over **L**	[8,10]
m_{EL}	20	$\mathrm{kg\,m^{-3}\,d^{-1}}$		**E** over **L**	[8,10]
m_{ME}	20	$\mathrm{kg\,m^{-3}\,d^{-1}}$		**M** over **E**	[8,10]
γ_M	$1.5 \cdot 10^{-7}$	$\mathrm{kg\,m^{-1}\,d^{-2}}$	Tensor coefficient	Microalgae **M**	[8,10]
γ_E	$1.5 \cdot 10^{-7}$	$\mathrm{kg\,m^{-1}\,d^{-2}}$		Extra-cellular matrix **E**	[8,10]
ρ	1.025	$\mathrm{kg\,L^{-1}}$	Volumetric mass density of liquid		

S3 Table F. Estimated values of the friction coefficients and tensor coefficients in the force balance equation Eq. 4

References

1. Bernard O, Sciandra A, and Madani S. Multimodel analysis of the response of the coccolithophore Emiliania huxleyi to an elevation of pCO2 under nitrate limitation. Ecological Modelling 2008;211:324 –338.
2. Bernard O, Boulanger AC, Bristeau MO, and Sainte-Marie J. A 2D model for hydrodynamics and biology coupling applied to algae growth simulations. ESAIM: Mathematical Modelling and Numerical Analysis 2013;47:1387–412.
3. Bernard O. Hurdles and challenges for modelling and control of microalgae for CO2 mitigation and biofuel production. Journal of Process Control 2011;21:1378–89.
4. Edmundson SJ and Huesemann MH. The dark side of algae cultivation: characterizing night biomass loss in three photosynthetic algae, Chlorella sorokiniana, Nannochloropsis salina and Picochlorum sp. Algal research 2015;12:470–6.
5. Mairet F, Bernard O, Masci P, Lacour T, and Sciandra A. Modelling neutral lipid production by the microalga Isochrysis aff. galbana under nitrogen limitation. Bioresource technology 2011;102:142–9.
6. Sohm JA, Edwards BR, Wilson BG, and Webb EA. Constitutive extracellular polysaccharide (EPS) production by specific isolates of Crocosphaera watsonii. Frontiers in microbiology 2011;2:229.
7. Serra-Maia R, Bernard O, Gonçalves A, Bensalem S, and Lopes F. Influence of temperature on Chlorella vulgaris growth and mortality rates in a photobioreactor. Algal research 2016;18:352–9.
8. Clarelli F, Di Russo C, Natalini R, and Ribot M. A fluid dynamics model of the growth of phototrophic biofilms. Journal of mathematical biology 2013;66:1387–408.
9. Stomp M, Huisman J, Stal LJ, and Matthijs HC. Colorful niches of phototrophic microorganisms shaped by vibrations of the water molecule. The ISME journal 2007;1:271–82.
10. Clarelli F, Di Russo C, Natalini R, and Ribot M. A fluid dynamics multidimensional model of biofilm growth: stability, influence of environment and sensitivity. Mathematical medicine and biology: a journal of the IMA 2016;33:371–95.
11. Zippel B, Rijstenbil J, and Neu TR. A flow-lane incubator for studying freshwater and marine phototrophic biofilms. Journal of Microbiological Methods 2007;70:336 –345.
12. Costache T, Fernández FGA, Morales M, Fernández-Sevilla J, Stamatin I, and Molina E. Comprehensive model of microalgae photosynthesis rate as a function of culture conditions in photobioreactors. Applied microbiology and biotechnology 2013;97:7627–37.
13. Lange O, Meyer A, and Budel B. Net photosynthesis activation of a desiccated cyano-bacterium without liquid water in high air humidity alone. Experiments with Microcoleus sociatus isolated from a desert soil crust. Functional Ecology 1994:52–7.
14. Novak JT and Brune DE. Inorganic carbon limited growth kinetics of some freshwater algae. Water research 1985;19:215–25.
15. Henze M, Grady C, Gujer W, Marais G, and Matsuo T. A general model for single-sludge wastewater treatment systems. Water Research 1987;21:505 –515.

16. Wolf G, Picioreanu C, and Loosdrecht MC van. Kinetic modeling of phototrophic biofilms: the PHOBIA model. Biotechnology and bioengineering 2007;97:1064–79.
17. Zeebe RE. On the molecular diffusion coefficients of dissolved CO2, HCO3-, and CO32-and their dependence on isotopic mass. Geochimica et Cosmochimica Acta 2011;75:2483–98.
18. Karimi A, Golbabaei F, Mehrnia MR, et al. Oxygen mass transfer in a stirred tank bioreactor using different impeller configurations for environmental purposes. Iranian journal of environmental health science & engineering 2013;10:1–9.
19. Polizzi B, Bernard O, and Ribot M. A time-space model for the growth of microalgae biofilms for biofuel production. Journal of Theoretical Biology 2017;432:55 –79.
20. Xiao R and Zheng Y. Overview of microalgal extracellular polymeric substances (EPS) and their applications. Biotechnology Advances 2016;34:1225–44.
