# Supplementary material for: Understanding photosynthetic biofilm productivity and structure through 2D simulation
Source: PLoS Comput Biol. 2022 Apr 4;18(4):e1009904. doi: 10.1371/journal.pcbi.1009904 (PMC9037940; doi:10.1371/journal.pcbi.1009904)
Supplement: S4 Text — (PDF) [file pcbi.1009904.s004.pdf]

Understanding photosynthetic biofilm productivity and structure through 2D simulation

Bastien Polizzi[1*], Andrea Fanesi[2], Filipa Lopes[2], Magali Ribot[3], Olivier Bernard[4,5],

1 Laboratoire de Mathématiques de Besançon, Université Bourgogne Franche-Comté, CNRS UMR-6623, 16, route de Gray, 25030 Besançon Cedex, France
2 LGPM, CentraleSupélec, 3, rue Joliot-Curie 91192 Gif-Sur-Yvette Cedex, France
3 IDP, Université d'Orléans, CNRS, UMR CNRS 7013, rue de Chartres, BP 6759, F-45067 Orléans Cedex 2, France
4 BIOCORE, Inria Sophia Antipolis Méditerranée Research Centre, Valbonne, France
5 LOV-UPMC-CNRS, UMR 7093, Station Zoologique, Villefranche-sur-mer, France

Supporting information

S4 Numerical scheme

This appendix presents the numerical scheme used to discretize the full set of equations. This scheme follows, for the spatial discretization, the strategy of [1] for a similar model, that is to say some finite-difference scheme derived from relaxation techniques. In this article, the authors dealt with the two following issues: the computation of the velocities in the case of a vanishing phase and the computation of the pressure term. However, we have to face another difficulty here: the mass exchanges between components are non linear and stiff, so we use some explicit-implicit treatment of the source terms of the mass balance equations in order to preserve the non-negativity of the solutions.

We consider the two dimensional domain $\Omega = [0, L_x] \times [0, L_z]$ of width L_x and height L_z. The extension of the following scheme to the 3D case is straightforward, but in this paper we focus our attention on the two dimensional case. We denote by Δx (resp. Δz) the space step in the x (resp. z) direction and we use a regular grid on Ω, namely the discretization points are (x_i, z_j) where $x_i = i\Delta x$ for $1 \leq i \leq N_x$ and $z_i = j\Delta z$ for $1 \leq j \leq N_z$. The kth time step is denoted by Δt_k and the time discretizations are therefore equal to $t^n = \sum_{k=1}^{n} \Delta t_k$. Computational parameters are given at Table A.

Name	Value	Unit	Interpretation
L_x	$3 \cdot 10^{-3}$	m	length of the computational domain in the x-direction
L_z	$3 \cdot 10^{-3}$	m	length of the computational domain in the z-direction
N_x	400		Number of discretization points in the x-direction
N_z	400		Number of discretization points in the z-direction

S4 Table A. Parameter values for the numerical discretization

We begin with rewriting the full system of S1 Text under the following form:

$$\partial_t U + \nabla_X \cdot \mathcal{F}(U,W) = \mathbf{\Gamma}(U) + \nabla_X \cdot \left(M_D L \nabla_X \left(\frac{U}{L}\right)\right), \tag{10a}$$

$$\partial_t W + \nabla_X \cdot \mathcal{G}(U,W) = \mathcal{G}_{\mathbf{I}}(U,W) + \mathcal{G}_{\mathbf{T}}(U,W) + \mathcal{G}_{\mathbf{P}}(U), \tag{10b}$$

$$L = 1 - A - N - E, \tag{10c}$$

$$\nabla_X \cdot ((A+N)\mathbf{v}_M + E\mathbf{v}_E + L\mathbf{v}_L) = \frac{\Gamma_A + \Gamma_N}{\rho_M} + \frac{\Gamma_E}{\rho_E} + \frac{\Gamma_L}{\rho_L}, \tag{10d}$$

that is to say one equation for the mass balances, one equation for the force balances, the volume condition and the incompressibility constraint. Here, U is a vector containing the mass fractions, W is a vector containing the three velocities, $\mathcal{F}$ (resp. $\mathcal{G}$) is the flux of vector U (resp. of vector W), M_D is the diffusion matrix for U, $\mathbf{\Gamma}(U)$ is the mass exchanges source term in the mass balance equations. The source terms in the force balance equations are split into three parts: the pressure part $\mathcal{G}_{\mathbf{P}}(U)$, the interactions term $\mathcal{G}_{\mathbf{I}}(U,W)$ and the mass exchanges term $\mathcal{G}_{\mathbf{T}}(U,W)$, that is to say:

$$U = \begin{pmatrix} A \\ N \\ E \\ SL \\ CL \\ OL \end{pmatrix} \in \mathbb{R}^6, \qquad W = \begin{pmatrix} (A+N)\mathbf{v}_M \\ E\mathbf{v}_E \\ L\mathbf{v}_L \end{pmatrix} = \begin{pmatrix} (A+N)v_{M,x} \\ (A+N)v_{M,z} \\ Ev_{E,x} \\ Ev_{E,z} \\ Lv_{L,x} \\ Lv_{L,z} \end{pmatrix} \in \mathbb{R}^6,$$

$$\mathcal{F}(U,W) = \begin{pmatrix} A\mathbf{v}_M \\ N\mathbf{v}_M \\ E\mathbf{v}_E \\ SL\mathbf{v}_L \\ CL\mathbf{v}_L \\ OL\mathbf{v}_L \end{pmatrix} = \begin{pmatrix} Av_{M,x} & Av_{M,z} \\ Nv_{M,x} & Nv_{M,z} \\ Ev_{E,x} & Ev_{E,z} \\ SLv_{L,x} & SLv_{L,z} \\ CLv_{L,x} & CLv_{L,z} \\ OLv_{L,x} & OLv_{L,z} \end{pmatrix} \in \mathbb{R}^{6\times 2},$$

$$\mathbf{\Gamma}(U) = \begin{pmatrix} \Gamma_A \cdot \rho_M^{-1} \\ \Gamma_N \cdot \rho_M^{-1} \\ \Gamma_E \cdot \rho_E^{-1} \\ \Gamma_S \cdot \rho_L^{-1} \\ \Gamma_C \cdot \rho_L^{-1} \\ \Gamma_O \cdot \rho_L^{-1} \end{pmatrix} \in \mathbb{R}^6, \quad M_D = \begin{pmatrix} 0 & & & & & \\ & 0 & & & & \\ & & 0 & & & \\ & & & 0 & & \\ & & & & D_S & \\ & & & & & D_C & \\ & & & & & & D_O \end{pmatrix} \in \mathcal{M}_{6,6}(\mathbb{R}),$$

$$\mathcal{G}(U,W) = \begin{pmatrix} (A+N)(\mathbf{v}_M \otimes \mathbf{v}_M + \gamma_M \mathbf{I}_2) \\ E(\mathbf{v}_E \otimes \mathbf{v}_E + \gamma_E \mathbf{I}_2) \\ L\mathbf{v}_L \otimes \mathbf{v}_L \end{pmatrix} \in \mathbb{R}^{6\times 2}$$

$$\mathcal{G}_{\mathbf{P}}(U) = \begin{pmatrix} -(A+N)\nabla_X P \\ -E\nabla_X P \\ -L\nabla_X P \end{pmatrix} = \begin{pmatrix} -(A+N)\partial_x P \\ -(A+N)\partial_z P \\ -E\partial_x P \\ -E\partial_z P \\ -L\partial_x P \\ -L\partial_z P \end{pmatrix} \in \mathbb{R}^6,$$

$$\mathcal{G}_{\mathbf{I}}(U,W) = \begin{pmatrix} -\dfrac{m_{ML}}{\rho_M}(\mathbf{v}_M - \mathbf{v}_L) - \dfrac{m_{ME}}{\rho_M}(\mathbf{v}_M - \mathbf{v}_E) \\ -\dfrac{m_{EL}}{\rho_E}(\mathbf{v}_E - \mathbf{v}_L) + \dfrac{m_{ME}}{\rho_E}(\mathbf{v}_M - \mathbf{v}_E) \\ \dfrac{m_{ML}}{\rho_L}(\mathbf{v}_M - \mathbf{v}_L) + \dfrac{m_{EL}}{\rho_L}(\mathbf{v}_E - \mathbf{v}_L) \end{pmatrix} \in \mathbb{R}^6,$$

$$\mathcal{G}_{\mathbf{T}}(U,W) = \begin{pmatrix} \dfrac{\Gamma_A + \Gamma_N}{\rho_M}\mathbf{v}_M \\ \dfrac{\Gamma_E}{\rho_E}\mathbf{v}_E \\ -\frac{1}{\rho_L}((\Gamma_A + \Gamma_N)\mathbf{v}_M + \Gamma_E \mathbf{v}_E) \end{pmatrix} \in \mathbb{R}^6.$$

In order to define the numerical approximation, we rewrite the fluxes $\mathcal{F}$ as $\mathcal{F} = (\mathcal{F}^x, \mathcal{F}^z)$ with

$$\mathcal{F}^x(U,W) = \begin{pmatrix} Av_{M,x} \\ Nv_{M,x} \\ Ev_{E,x} \\ SLv_{L,x} \\ CLv_{L,x} \\ OLv_{L,x} \end{pmatrix} \in \mathbb{R}^6, \qquad \text{and} \qquad \mathcal{F}^z(U,W) = \begin{pmatrix} Av_{M,z} \\ Nv_{M,z} \\ Ev_{E,z} \\ SLv_{L,z} \\ CLv_{L,z} \\ OLv_{L,z} \end{pmatrix} \in \mathbb{R}^6.$$

and $\mathcal{G}$ as $\mathcal{G} = (\mathcal{G}^x, \mathcal{G}^z)$ with

$$\mathcal{G}^x(U,W) = \begin{pmatrix} (A+N)(v_{M,x}^2 + \gamma_M) \\ (A+N)v_{M,x}v_{M,z} \\ E(v_{E,x}^2 + \gamma_E) \\ Ev_{E,x}v_{E,z} \\ Lv_{L,x}^2 \\ Lv_{L,x}v_{L,z} \end{pmatrix} \in \mathbb{R}^6, \text{ and } \mathcal{G}^z(U,W) = \begin{pmatrix} (A+N)v_{M,x}v_{M,z} \\ (A+N)(v_{M,z}^2 + \gamma_M) \\ Ev_{E,x}v_{E,z} \\ E(v_{E,z}^2 + \gamma_E) \\ Lv_{L,x}v_{L,z} \\ Lv_{L,z}^2 \end{pmatrix} \in \mathbb{R}^6.$$

We denote by $U_{i,j}^n$ and $W_{i,j}^n$ the discrete approximations of $U(t^n, x_i, z_j)$ and $W(t^n, x_i, z_j)$.

S4.1 Numerical approximation of mass balance equations

The first step consists in solving equation (10a), using an explicit discretization based on a relaxation technique, presented in [2] and used in [1], for the flux term; a mixed strategy for the diffusion term and an explicit method for the source term. More precisely for the diffusion, U is treated semi-implicitly using Crank-Nicolson method, whereas L is taken explicitly. The spatial discretization is done with a finite difference method which involves numerical approximations of L on the edges of the mesh cells; they are interpolated as the mean values of L on the cells sharing this edge, namely we use the approximations $L_{i+\frac{1}{2},j} = \frac{1}{2}(L_{i+1,j} + L_{i,j})$ and $L_{i,j+\frac{1}{2}} = \frac{1}{2}(L_{i,j+1} + L_{i,j})$. The source term can be treated explicitly, since we need to take the time step small enough.

We obtain therefore a scheme of the form:

$$
\begin{aligned}
U_{i,j}^{n+1} &- \frac{\Delta t}{2\Delta x^2} M_D \left(L_{i+\frac{1}{2},j}^n \left(\frac{U_{i+1,j}^{n+1}}{L_{i+1,j}^n} - \frac{U_{i,j}^{n+1}}{L_{i,j}^n} \right) - L_{i-\frac{1}{2},j}^n \left(\frac{U_{i,j}^{n+1}}{L_{i,j}^n} - \frac{U_{i-1,j}^{n+1}}{L_{i-1,j}^n} \right) \right) \\
&- \frac{\Delta t}{2\Delta z^2} M_D \left(L_{i,j+\frac{1}{2}}^n \left(\frac{U_{i,j+1}^{n+1}}{L_{i,j+1}^n} - \frac{U_{i,j}^{n+1}}{L_{i,j}^n} \right) - L_{i,j-\frac{1}{2}}^n \left(\frac{U_{i,j}^{n+1}}{L_{i,j}^n} - \frac{U_{i,j-1}^{n+1}}{L_{i,j-1}^n} \right) \right) \\
&= U_{i,j}^n + \Delta t \mathbf{\Gamma}(U_{i,j}^n) - \frac{\Delta t}{2\Delta x} \left(\mathcal{F}^x(U_{i+1,j}^n, W_{i+1,j}^n) - \mathcal{F}^x(U_{i-1,j}^n, W_{i-1,j}^n) \right) \\
&\quad - \frac{\Delta t}{2\Delta z} \left(\mathcal{F}^z(U_{i,j+1}^n, W_{i,j+1}^n) - \mathcal{F}^z(U_{i,j-1}^n, W_{i,j-1}^n) \right) \\
&\quad + \frac{\Delta t}{2\Delta x^2} M_D \left(L_{i+\frac{1}{2},j}^n \left(\frac{U_{i+1,j}^n}{L_{i+1,j}^n} - \frac{U_{i,j}^n}{L_{i,j}^n} \right) - L_{i-\frac{1}{2},j}^n \left(\frac{U_{i,j}^n}{L_{i,j}^n} - \frac{U_{i-1,j}^n}{L_{i-1,j}^n} \right) \right) \\
&\quad + \frac{\Delta t}{2\Delta z^2} M_D \left(L_{i,j+\frac{1}{2}}^n \left(\frac{U_{i,j+1}^n}{L_{i,j+1}^n} - \frac{U_{i,j}^n}{L_{i,j}^n} \right) - L_{i,j-\frac{1}{2}}^n \left(\frac{U_{i,j}^n}{L_{i,j}^n} - \frac{U_{i,j-1}^n}{L_{i,j-1}^n} \right) \right) \\
&\quad + \lambda^n \frac{\Delta t}{4\Delta x} \left(U_{i+1,j}^n - 2U_{i,j}^n + U_{i-1,j}^n \right) + \lambda^n \frac{\Delta t}{4\Delta z} \left(U_{i,j+1}^n - 2U_{i,j}^n + U_{i,j-1}^n \right)
\end{aligned}
$$

where the numerical velocity λ^n is common to the mass balance equations and to the force balance equations and is equal to the maximum of the eigenvalues of the Jacobian matrix of the fluxes $\begin{pmatrix} \mathcal{F} \\ \mathcal{G} \end{pmatrix}$, that is to say:

$$
\lambda^n = \max_{i,j} \left\{ 2\|v_{L,i,j}^n\|_\infty, \|v_{M,i,j}^n\|_\infty + \sqrt{\frac{\gamma_M}{\rho_M}}, \|v_{E,i,j}^n\|_\infty + \sqrt{\frac{\gamma_E}{\rho_E}} \right\}.
$$

For stability reasons, the time step Δt^n is computed at each time step such that the stability condition $\lambda^n \dfrac{\Delta t^n}{\max(\Delta x, \Delta z)} \leq 1$ is satisfied.

The overall strategy at time step t^{n+1} is the following: we first obtain the volume fractions $(SL)^{n+1}$, $(CL)^{n+1}$ and $(OL)^{n+1}$ and the remaining components of U^{n+1}, that is to say A^{n+1}, N^{n+1} and E^{n+1}. Finally, an approximation of the volume fraction of liquid is given thanks to condition (10c).

S4.2 Numerical approximation of force balance equations

Dealing with the computation of force balance equations, we face two difficulties: vanishing phases and computation of the pressure term.

Firstly, force balance equations give the evolution of the momentum of each component, whereas the friction forces depend on the velocities. As a consequence, when one of the phases is vanishing, it is not clear how to define its velocity, that is needed to approximate the friction forces at the following time step. Note that, in a biological context, situations where for example $L = 1$ and $A = N = E = 0$ are relevant and we cannot claim to be far from vacuum, as can be done in a physical context. Therefore, in order to compute the velocities, we use an implicit-explicit time discretization strategy for the momentum equations, where the interaction forces term $\mathcal{G}_{\mathbf{I}}(U, W)$ is treated implicitly, see [1].

Secondly, to compute the velocities, we need to know the gradient of the hydrostatic pressure, which is another unknown of the system. A natural approach consists in finding an equation verified by P, by taking the divergence of the sum of the momentum equations and by using eq. (10d). However, this method, which is known to

be inefficient, leads to an elliptic equation for P with a non-unique solution. To overcome this difficulty we use a splitting approach which is basically an adaptation of the Chorin-Temam projection method [3,4], see again [1]. This method uses a projection-correction approach: first, we compute an approximation of the velocities using the force balance equations without the pressure terms; then, using the predicted velocities and the average incompressibility constraint, we compute the pressure as the solution of an elliptic equation. Finally, the velocities are corrected thanks to the value of $\nabla_X P$.

Let us recall that the vector U^{n+1} is computed at a previous step. We now give some details on the scheme for the force balance equations computation.

First, the projection step enables us to estimate some predicted values $V^{n+\frac{1}{2}}$ for the velocities. We denote by

$$V_{i,j}^{n+\frac{1}{2}} = \begin{pmatrix} v_{M,x,i,j}^{n+\frac{1}{2}} \\ v_{M,z,i,j}^{n+\frac{1}{2}} \\ v_{E,x,i,j}^{n+\frac{1}{2}} \\ v_{E,z,i,j}^{n+\frac{1}{2}} \\ v_{L,x,i,j}^{n+\frac{1}{2}} \\ v_{L,z,i,j}^{n+\frac{1}{2}} \end{pmatrix}$$

and this first step of the scheme writes as:

$$\begin{aligned} \mathcal{M}_{i,j}^{n+1} V_{i,j}^{n+\frac{1}{2}} = W_{i,j}^n &- \frac{\Delta t}{2\Delta x}\big(\mathcal{G}^x\big(U_{i+1,j}^n, W_{i+1,j}^n\big) - \mathcal{G}^x\big(U_{i-1,j}^n, W_{i-1,j}^n\big)\big) \\ &- \frac{\Delta t}{2\Delta z}\big(\mathcal{G}^z\big(U_{i,j+1}^n, W_{i,j+1}^n\big) - \mathcal{G}^z\big(U_{i,j-1}^n, W_{i,j-1}^n\big)\big) \\ &+ \lambda^n \frac{\Delta t}{4\Delta x}\big(W_{i+1,j}^n - 2W_{i,j}^n + W_{i-1,j}^n\big) \\ &+ \lambda^n \frac{\Delta t}{4\Delta z}\big(W_{i,j+1}^n - 2W_{i,j}^n + W_{i,j-1}^n\big) \\ &+ \Delta t \mathcal{G}_{\mathbf{T}}\big(U_{i,j}^{n+1}, W_{i,j}^n\big) \end{aligned} \tag{11}$$

where

$$\mathcal{G}_{\mathbf{T}}\big(U^{n+1}, W^n\big) = \begin{pmatrix} \dfrac{\Gamma_A^{n+1} + \Gamma_N^{n+1}}{\rho_M} v_{M,x}^n \\ \dfrac{\Gamma_A^{n+1} + \Gamma_N^{n+1}}{\rho_M} v_{M,z}^n \\ \dfrac{\Gamma_E^{n+1}}{\rho_E} v_{E,x}^n \\ \dfrac{\Gamma_E^{n+1}}{\rho_E} v_{E,z}^n \\ -\dfrac{1}{\rho_L}\big((\Gamma_A^{n+1} + \Gamma_N^{n+1}) v_{M,x}^n + \Gamma_E^{n+1} v_{E,x}^n\big) \\ -\dfrac{1}{\rho_L}\big((\Gamma_A^{n+1} + \Gamma_N^{n+1}) v_{M,z}^n + \Gamma_E^{n+1} v_{E,z}^n\big) \end{pmatrix},$$

$$\mathcal{M}_{i,j}^{n+1} = \begin{pmatrix} \Lambda_1 & 0 & -\frac{\Delta t}{\rho_M} m_{ME} & 0 & -\frac{\Delta t}{\rho_M} m_{ML} & 0 \\ 0 & \Lambda_1 & 0 & -\frac{\Delta t}{\rho_M} m_{ME} & 0 & -\frac{\Delta t}{\rho_M} m_{ML} \\ -\frac{\Delta t}{\rho_E} m_{ME} & 0 & \Lambda_2 & 0 & -\frac{\Delta t}{\rho_E} m_{EL} & 0 \\ 0 & -\frac{\Delta t}{\rho_E} m_{ME} & 0 & \Lambda_2 & 0 & -\frac{\Delta t}{\rho_E} m_{EL} \\ -\frac{\Delta t}{\rho_L} m_{ML} & 0 & -\frac{\Delta t}{\rho_L} m_{EL} & 0 & \Lambda_3 & 0 \\ 0 & -\frac{\Delta t}{\rho_L} m_{ML} & 0 & -\frac{\Delta t}{\rho_L} m_{EL} & 0 & \Lambda_3 \end{pmatrix},$$

$$\Lambda_1 = A_{i,j}^{n+1} + N_{i,j}^{n+1} + \frac{\Delta t}{\rho_M}(m_{ML} + m_{ME}),$$
$$\Lambda_2 = E_{i,j}^{n+1} + \frac{\Delta t}{\rho_E}(m_{EL} + m_{ME}),$$
$$\Lambda_3 = L_{i,j}^{n+1} + \frac{\Delta t}{\rho_L}(m_{ML} + m_{EL}).$$

Note that the matrix $\mathcal{M}$ contains also the coefficients of the interaction terms, which are treated implicitly, and that this matrix can be computed thanks to the knowledge of U^{n+1}. Moreover, we can compute the determinant of $\mathcal{M}$, namely

$$\begin{aligned} \det \mathcal{M} = \Big(&(A+N)EL \\ &+ \Delta t \left(\frac{m_{ML}}{\rho_L}(A+N)E + \frac{m_{EL}}{\rho_L}(A+N)E + \frac{m_{EL}}{\rho_E}(A+N)L \right. \\ &\left. + \frac{m_{ME}}{\rho_E}(A+N)L + \frac{m_{ML}}{\rho_M}EL + \frac{m_{ME}}{\rho_M}EL \right) \\ &+ \Delta t^2 (m_{EL} m_{ML} + m_{ME} m_{ML} + m_{ME} m_{EL}) \\ &\times \left(\frac{A+N}{\rho_E \rho_L} + \frac{E}{\rho_M \rho_L} + \frac{L}{\rho_M \rho_E} \right) \Big)^2, \end{aligned}$$

which is a polynomial of degree 2 in Δt with positive coefficients. Since all the friction coefficients are strictly positive and since the constraint $A + N + E + L = 1$, with A, N, E, L positive, is satisfied, the leading coefficient of $\det \mathcal{M}$ is non zero. So, for all Δt strictly positive, $\det \mathcal{M}$ does not vanish and linear system (11) has a unique solution.

Then in the second step of splitting we have to solve

$$\partial_t(\phi \mathbf{v}_\phi) = -\frac{\phi}{\rho_\phi} \nabla_X P \tag{12}$$

for $\phi = M, E, L$ in the interval $[t, t + \Delta t^n]$ with initial data $\phi^{n+1} \mathbf{v}_\phi^{n+\frac{1}{2}}$. The discrete approximation of these equations for $\phi = M, E, L$ is given by

$$\phi^{n+1} \mathbf{v}_\phi^{n+1} - \phi^{n+1} \mathbf{v}_\phi^{n+\frac{1}{2}} = -\Delta t \frac{\phi^{n+1}}{\rho_\phi} \nabla_X P^{n+1}.$$

Then taking the divergence of the sum of these equations over ϕ we get

$$\begin{aligned} &\nabla_X \cdot \big((A^{n+1} + N^{n+1}) \mathbf{v}_M^{n+1} + E^{n+1} \mathbf{v}_E^{n+1} + L^{n+1} \mathbf{v}_L^{n+1} \big) \\ &- \nabla_X \cdot \Big((A^{n+1} + N^{n+1}) \mathbf{v}_M^{n+\frac{1}{2}} + E^{n+1} \mathbf{v}_E^{n+\frac{1}{2}} + L^{n+1} \mathbf{v}_L^{n+\frac{1}{2}} \Big) \\ &\quad = -\Delta t \nabla_X \cdot \left(\left(\frac{A^{n+1} + N^{n+1}}{\rho_M} + \frac{E^{n+1}}{\rho_E} + \frac{L^{n+1}}{\rho_L} \right) \nabla_X P^{n+1} \right). \end{aligned}$$

Now using the discrete approximation of the incompressibility constraint (10d), we deduce that P is solution of an elliptic equation with non constant coefficients:

$$\begin{aligned}
&\Delta t \nabla_X \cdot \left(\left(\frac{(A^{n+1} + N^{n+1})}{\rho_M} + \frac{E^{n+1}}{\rho_E} + \frac{L^{n+1}}{\rho_L} \right) \nabla_X P^{n+1} \right) = \\
&\nabla_X \cdot \left((A^{n+1} + N^{n+1}) \mathbf{v}_M^{n+\frac{1}{2}} + E^{n+1} \mathbf{v}_E^{n+\frac{1}{2}} + L^{n+1} \mathbf{v}_L^{n+\frac{1}{2}} \right) \\
&- \frac{\Gamma_A^{n+1} + \Gamma_N^{n+1}}{\rho_M} - \frac{\Gamma_E^{n+1}}{\rho_E} - \frac{\Gamma_L^{n+1}}{\rho_L}
\end{aligned} \tag{13}$$

This equation is complemented with Neumann boundary conditions on P. This boundary condition can be directly deduced from boundary conditions on the velocities and equation (12) on the boundary $\Gamma_{\neq L_z}$. For $z = L_z$, we need to note that, in this step, $\mathbf{v}_\phi$ does not change since none of the volume or mass fractions are modified, so using equation (12) we also get Neumann boundary condition for the pressure on $z = L_z$. However, with Neumann boundary conditions on P, equation (13) does not have a unique solution. In practice, we choose the solution for which the average value of P is null, that is to say the solution satisfying

$$\int_{[0,L_x]\times[0,L_z]} P(t,x,z)\ dx\ dz = 0.$$

Finally in order to get a symmetric system we rather solve the minimisation problem

$$\begin{aligned}
&\Delta t \nabla_X \cdot \left(\left(\frac{A^{n+1} + N^{n+1}}{\rho_M} + \frac{E^{n+1}}{\rho_E} + \frac{L^{n+1}}{\rho_L} \right) \nabla_X P^{n+1} \right) + \mu = \\
&\nabla_X \cdot \left((A^{n+1} + N^{n+1}) \mathbf{v}_M^{n+\frac{1}{2}} + E^{n+1} \mathbf{v}_E^{n+\frac{1}{2}} + L^{n+1} \mathbf{v}_L^{n+\frac{1}{2}} \right) \\
&- \frac{\Gamma_A^{n+1} + \Gamma_N^{n+1}}{\rho_M} - \frac{\Gamma_E^{n+1}}{\rho_E} - \frac{\Gamma_L^{n+1}}{\rho_L}, \\
&\int_{[0,L_x]\times[0,L_z]} P^{n+1}(x,z)\ dx\ dz = 0, \\
&\nabla_X P^{n+1}(x,z) = 0, \text{ on } \Gamma_{\neq L_z}\ \nabla_X P^{n+1}(x, L_z) = 0,\ x \in [0, L_x]
\end{aligned}$$

with μ the Lagrange multiplier associated to the constraint $\int_{[0,L_x]\times[0,L_z]} P^{n+1}\ dx\ dz = 0$. This system is discretized using classical centered finite difference method and leads to the resolution of a linear symmetric system.

Finally since the volume fractions do not change in this step, we update velocities as follows:

$$\begin{aligned}
\mathbf{v}_M^{n+1} &= \mathbf{v}_M^{n+\frac{1}{2}} - \frac{\Delta t}{\rho_M} (\nabla_X P)^{n+1}, \\
\mathbf{v}_E^{n+1} &= \mathbf{v}_E^{n+\frac{1}{2}} - \frac{\Delta t}{\rho_E} (\nabla_X P)^{n+1}, \\
\mathbf{v}_L^{n+1} &= \mathbf{v}_L^{n+\frac{1}{2}} - \frac{\Delta t}{\rho_L} (\nabla_X P)^{n+1}.
\end{aligned}$$

References

1. Clarelli F, Di Russo C, Natalini R, and Ribot M. A fluid dynamics model of the growth of phototrophic biofilms. Journal of mathematical biology 2013;66:1387–408.

2. Aregba-Driollet D and Natalini R. Discrete kinetic schemes for multidimensional systems of conservation laws. SIAM Journal on Numerical Analysis 2000;37:1973–2004.
3. Temam R. Une méthode d'approximation de la solution des équations de Navier-Stokes. Bulletin de la Société Mathématique de France 1968;96:115–52.
4. Chorin AJ. Numerical solution of the Navier-Stokes equations. Mathematics of computation 1968;22:745–62.
