# Supplementary material for: Understanding photosynthetic biofilm productivity and structure through 2D simulation
Source: PLoS Comput Biol. 2022 Apr 4;18(4):e1009904. doi: 10.1371/journal.pcbi.1009904 (PMC9037940; doi:10.1371/journal.pcbi.1009904)
Supplement: S5 Text — (PDF) [file pcbi.1009904.s005.pdf]

Understanding photosynthetic biofilm productivity and structure through 2D simulation

Bastien Polizzi[1*], Andrea Fanesi[2], Filipa Lopes[2], Magali Ribot[3], Olivier Bernard[4,5],

1 Laboratoire de Mathématiques de Besançon, Université Bourgogne Franche-Comté, CNRS UMR-6623, 16, route de Gray, 25030 Besançon Cedex, France
2 LGPM, CentraleSupélec, 3, rue Joliot-Curie 91192 Gif-Sur-Yvette Cedex, France
3 IDP, Université d'Orléans, CNRS, UMR CNRS 7013, rue de Chartres, BP 6759, F-45067 Orléans Cedex 2, France
4 BIOCORE, Inria Sophia Antipolis Méditerranée Research Centre, Valbonne, France
5 LOV-UPMC-CNRS, UMR 7093, Station Zoologique, Villefranche-sur-mer, France

Supporting information

S5 Photosynthesis rate insight for multi-spot colony simulation

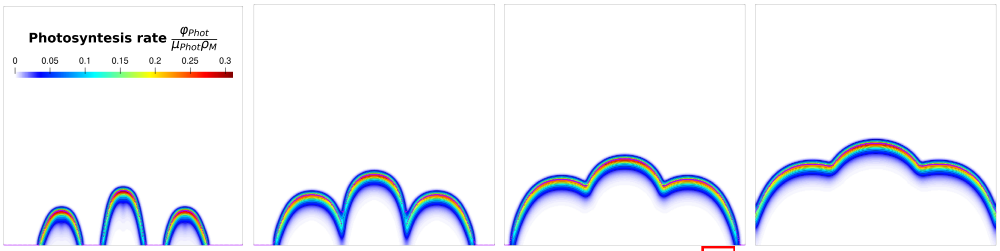

S5 Fig A. Profile of the photosynthesis law (ie. φ_{Phot}, see S2 for the detailed mathematical expression) at times $t = 5$, $t = 10$, $t = 15$ and $t = 20$ days for a biofilm starting from a multi-spot colony. The purple dotted line represents the biofilm front defined as the largest value for the biofilm gradient.

Fig A represents the photosynthesis rate at different times in the case where the simulation is initialised with a multi-spot colony, see article's section 3.3. As for the single spot colony, it can be observed that photosynthesis happens only on the outer layer of the biofilm. Moreover, the thickness of the active layer remains in the range $75 \pm 7.5\mu m$ while the biomass below this front remains inactive. Again, as in the simulation starting from a single spot colony, light is the main limiting factor for photosynthesis.
